# Targeted inhibition of DHODH is synergistic with BCL2 blockade in HGBCL with concurrent MYC and BCL2 rearrangement

**DOI:** 10.1186/s12885-024-12534-w

**Published:** 2024-06-25

**Authors:** Lin Liu, Wenbin Mo, Miao Chen, Yi Qu, Pingping Wang, Ying Liang, Xiaojing Yan

**Affiliations:** 1 Department of Hematology, The First Affiliated Hospital of China Medical University, Shenyang, 110001 China; 2 No. 155, North Nanjing Road, Heping District, Shenyang, 110001 China

**Keywords:** High-grade B-cell lymphoma, DHODH inhibitor, MYC, BCL2, Venetoclax

## Abstract

High-grade B-cell lymphoma (HGBCL), the subtype of non-Hodgkin lymphoma, to be relapsed or refractory in patients after initial therapy or salvage chemotherapy. Dual dysregulation of MYC and BCL2 is one of the important pathogenic mechanisms. Thus, combined targeting of MYC and BCL2 appears to be a promising strategy. Dihydroorotate dehydrogenase (DHODH) is the fourth rate-limiting enzyme for the de novo biosynthesis of pyrimidine. It has been shown to be a potential therapeutic target for multiple diseases. In this study, the DHODH inhibitor brequinar exhibited growth inhibition, cell cycle blockade, and apoptosis promotion in HGBCL cell lines with MYC and BCL2 rearrangements. The combination of brequinar and BCL2 inhibitors venetoclax had a synergistic inhibitory effect on the survival of DHL cells through different pathways. Venetoclax could upregulate MCL-1 and MYC expression, which has been reported as a resistance mechanism of BCL2 inhibitors. Brequinar downregulated MCL-1 and MYC, which could potentially overcome drug resistance to venetoclax in HGBCL cells. Furthermore, brequinar could downregulate a broad range of genes, including ribosome biosynthesis genes, which might contribute to its anti-tumor effects. In vivo studies demonstrated synergetic tumor growth inhibition in xenograft models with brequinar and venetoclax combination treatment. These results provide preliminary evidence for the rational combination of DHODH and BCL2 blockade in HGBCL with abnormal MYC and BCL2.

**Supplementary Information:**

The online version contains supplementary material available at 10.1186/s12885-024-12534-w.

## Introduction

Diffuse large B-cell lymphoma (DLBCL) is characterized by significant clinical heterogeneity, distinct pathologic subtypes, morphologic variants, and gene expression profiles [1, 2]. Standard chemoimmunotherapy R-CHOP (rituximab, cyclophosphamide, doxorubicin, vincristine, and prednisone) could result in approximately 50 ∼ 60% cure rates [1]. However, a considerable proportion of patients relapse or become refractory. Among the factors associated with refractory or relapsed (R/R) DLBCL, abnormalities in MYC and BCL2 are particularly important. DLBCL with rearrangements of MYC, BCL2, and/or BCL6, known as “double-hit lymphoma” (DHL), has been defined as a new entity and renamed “high-grade B-cell lymphoma (HGBCL) with rearrangements of MYC and BCL2 and/or BCL6” [3, 4]. HGBCL is uncommon, accounting for 5 ∼ 7% of all DLBCL patients. The prognosis is extremely poor after treatment with R-CHOP and even intensive chemotherapy with stem cell transplantation [3, 5, 6]. Concomitant overexpression of MYC and BCL2 protein is independent of MYC/BCL2 rearrangement and is known as double-expressor lymphoma (DEL) [3, 4]. It is not a distinct entity in the current WHO classification but accounts for 20 ∼ 30% of all DLBCL cases and is characterized by poor outcomes [5]. Thus, it is urgent to explore novel approaches for treating the specific entity of HGBCL and DEL. With the rapid development of small molecule inhibitors, the future of HGBCL and DEL therapy will likely incorporate targeted therapy into current regimens. Combined inhibition of MYC and BCL2 might be a novel, effective therapeutic strategy.

Several drugs directly target BCL2, among which venetoclax is the most promising, showing anti-tumor activity in a xenograft model of DLBCL and impressive response in patients with chronic lymphocytic leukemia (CLL) and acute myeloid leukemia (AML) [7–9]. Although a short exposure time to venetoclax can trigger significant anti-tumor effects in DLBCL cells, the clinical efficacy of venetoclax in DLBCL is less promising [10]. Recently, a single-agent dose-escalation trial of venetoclax in R/R NHL reported an overall response rate (ORR) of 18% and a complete response (CR) rate of 12% in patients with DLBCL [11]. Therefore, the therapeutic application of venetoclax to HGBCL and DEL lymphoma requires further investigation.

Despite the well-established role of MYC protein in tumorgenesis, no direct MYC-targeted therapeutic agent has been successfully used in the clinical setting for lymphoma. Modulation of transcription/epigenetic regulators of MYC has been investigated, including inhibition of the Bromo and extraterminal domain (BET) family or the associated pathways [12, 13]. However, the clinical safety and efficacy of this approach require further research [14]. DHODH, a druggable enzyme playing a vital role in the de novo pyrimidine synthesis pathway, is located in the outer membrane of mitochondria and is coupled with the electron transport chain [15]. Recent data show that inhibition of DHODH downregulates MYC expression, which may provide a new strategy for MYC-targeted therapy [15].

To develop and assess new therapeutics, we investigated the effects of the DHODH inhibitor brequinar and the synthetic effects of brequinar and venetoclax in the HGBCL lymphoma cell lines and xenograft mice model.

## Materials and methods

### Cell lines

DB, SU-DHL4, SU-DHL2, and SU-DHL10 cells were obtained from the American Type Cell Collection (ATCC) and grown in RPMI-1640 (Sigma, USA) supplemented with 10% heat-inactivated fetal bovine serum (PAN, Germany), 100 units/mL penicillin, and 100 µg/mL streptomycin (Gibco, USA). Cell lines were maintained in 5% CO_2_ at 37 °C. MYC and BCL2 rearrangements were confirmed by fluorescence in situ hybridization (FISH).

### Reagents

Brequinar (HY-108,325) and venetoclax (HY-15,531) were purchased from MCE (USA). DHODH antibody (ab174288) was purchased from Abcam (England). Antibodies targeting P53 (SC6243), P21 (SC817), and BAX (SC20067) were purchased from Santa Cruz. BCL2 (4223), BIM (C34C5), c-MYC (5605), p-c-MYC (46,650 and 13,748), MCL-1 (D35A5), BCL-xL (2762), α-tubulin (3873), caspase family antibody kit (9929), NFκB antibody kit (55,764), JAK/STAT antibody kit (9799), and PI3K/AKT antibody kit (9655) were purchased from CST (USA). Antibodies targeting GAPDH (10,494) and histones (19,649) were purchased from Proteintech (USA). Antibodies targeting RPL26 (102,758), RPS27 (138,642), and MRPS-6 (118,709) were purchased from Absin (China). Secondary anti-rabbit and anti-mouse polyclonal antibodies were purchased from Absin (China). Z-VAD-FMK was purchased from Selleck (America).

### Cell proliferation assay

Cell proliferation was measured by the Cell Counting Kit-8 (CCK8, Dojindo Laboratories, Japan) assay according to the manufacturer’s instructions. The cell viability was calculated by the formula: Cell viability (%) = [OD (drug+) - OD (Blank)] / [OD (drug-) - OD (Blank)]×100%. The IC50 value or CI value was calculated using GraphPad Prism 7.0 (GraphPad Software, San Diego, CA, USA).

### Cell cycle and apoptosis assay

For the apoptosis experiment, the percentage of viable cells was determined with an Annexin V-PE apoptosis detection kit I (BD Pharmingen, San Diego, CA) according to the manufacturer’s instructions using a FACSCanto II cytometer (BD). Cells negative for staining were considered viable, and all results were normalized to the dimethyl sulfoxide (DMSO)-treated group, which was set as 100% viable cells (0% dead cells). Data were analyzed with FlowJo software V7.6.1 (FlowJo LLC, Ashland, OR). For the cell cycle experiment, cells were harvested and fixed at 4 °C with 70% ethanol in phosphate-buffered saline (PBS) overnight. Cell suspensions were then treated with 50 µg/mL RNase A (Sigma; St Louis, MO) for 2 h before being stained with 50 µg/mL propidium iodide. The percentages of cells in the G0/G1, S, and G2/M phases were determined by flow cytometric analysis (FACSCalibur, BD). The cell cycle distribution was analyzed using BD ModFitTM LT software (BD Biosciences, San Diego, CA). Each experiment was repeated in triplicate.

### RT-PCR

RNA was extracted from relevant cells, and cDNA was generated using SuperScript® III RT (Thermo Fisher) with oligo-dT primers. qRT-PCR was performed using Power SYBR® Green PCR Master Mix (Applied Biosystems) according to the manufacturer’s instructions. GAPDH expression was used as an internal control. PCR was performed in duplicate wells on an ABI 9700 thermocycler (Applied Biosystems, Foster City, CA) under the following cycling conditions: 95 °C for 10 min and 35 cycles of 95 °C for 15 s and 60 °C for 1 min. The results were analyzed with GraphPad Prism 7 and are expressed as N-fold differences according to the ΔΔCq method as follows: relative expression = 2^–∆ΔCt^, where ΔCt = Ct (target gene) - Ct (control gene) [16]. The primer sequences are listed in Supplementary Table 1.

### DHODH knockout

We utilized the CRISPR-Cas9 design site (crispr.mit.edu) to identify sgRNAs targeting the protein-coding region of the human DHODH gene. We chose a sgRNA (5’-CAGTCACGGGCTTTCAGTGG-3’) based on the efficiency and low frequency of off-target sites. The lentiviral plasmids CRISPRV2-Cas9-Puro and CRISPRV2-Cas9-sgRNA-Puro were purchased from Miaoling Plasmid Biomart, China. We infected SU-DHL4 cells using the lentivirus packaging method.

### C-MYC overexpression cell construction

The c-MYC-overexpressing lentivirus pLV-hef1a-Puro-WPRE-CMV-MYC (human, NM_002467-3Xflag) and empty vector pLV-hef1a-Puro-WPRE-CMV-MCS-3Xflag were purchased from Hesheng Gene Company (China). DB and SU-DHL4 cells were transfected with the virus according to the instructions.

### Assessment of brequinar and venetoclax synergy

Combination indexes (CIs) for combinations of brequinar and venetoclax were calculated using Compusyn (CombosynInc, Paramus, NJ) according to the Chou-Talalay algorithm. The median CIs for all assessed combinations are shown.

### RNA-sequencing (RNA-SEQ) analysis

#### RNA extraction

A total of 5*10^6^ cells were suspended in 1000 µL TRIzol Reagent (Life Technologies). Then, RNA was extracted using an RNeasy Micro Kit (Qiagen, Germany) according to the manufacturer’s protocol.

#### Illumina RNA sequencing

A total amount of 1 µg RNA per sample was used as input material for the RNA sample preparations. Sequencing libraries were generated using the NEBNext® UltraTM RNA Library Prep Kit for Illumina® (NEB, USA) following the manufacturer’s recommendations, and index codes were added to attribute sequences to each sample. See “Supplementary Materials and Methods” for further information.

#### Sequencing data analysis

Differential expression analysis of two groups (two biological replicates per condition) was performed using the DESeq2 R package (1.16.1). The resulting *P*-values were adjusted using Benjamini and Hochberg’s approach for controlling the false discovery rate. Gene Ontology (GO) enrichment analysis of differentially expressed genes was implemented using the cluster Profiler R package, in which gene length bias was corrected. The GO terms/KEGG pathways analysis screen criteria were evaluated by Padj < 0.05. The “significant genes” between venetoclax and control groups were limited, so we used |log Foldchange|>0 as a threshold for further analysis.

### Western blot analysis

Proteins were prepared according to the handbook of Protein Extraction Kit from KeyGEN BioTECH (China). A 40 µg protein sample from each group was separated by SDS-PAGE and transferred onto a nitrocellulose membrane (Millipore, USA). The protein expression was detected by specific antibodies combined with HRP-conjugated secondary antibodies (goat anti-rabbit or goat anti-mouse)(Abison, China). Images were taken using an Olympus IX-71 microscope controlled by DeltaVision SoftWoRx. The immunoblots were quantified using ImageJ software (National Institute of Mental Health, Bethesda, MD, USA). For some molecules with molecular weight very close to that of the loading control, we first adjust our sample loading volume based on loading control. Then we loaded each sample (the same sample we used to adjust the loading volume) with recorded volumes to test different molecules.

### Co-immunoprecipitation assay

Lysis buffer was prepared with NP40 and PMSF (100:1) (Solarbio, China). Cell lysates were precleared with 20 µl of Protein A + G Agarose (SC-2003, Santa Cruz, USA) for 3 h at 4 °C before incubating with anti-BCL2, anti-BAX, or normal IgG antibodies (all from CST) overnight at 4 °C. Then, the proteins bound to each antibody were precipitated with protein agarose A + G for 4 h at 4 °C before they were resolved and analyzed by western blotting.

### In vivo studies

Four- to six-week-old SCID- NOD mice (SPF Biotechnology, Beijing, China) were bred under specific pathogen-free conditions. SU-DHL-4 cells (5 × 10^6^ cells) were injected subcutaneously into the right flank of the mice. Treatment started when the tumors were about 50mm^3^, and the mice were divided randomly into four groups, receiving vehicles(control), venetoclax, brequinar, or a combination of venetoclax and brequinar, respectively. Diameters of the tumors were measured, and the volume was calculated according to the equation: Tumor volume(mm^3^) =[Length(mm) × Width^2^(mm)]/2. Venetoclax (VEN) was administered at 50 mg/kg once daily by oral gavage in 60% phosal-50PG, 30% polyethylene glycol-400, and 10% ethanol [17]. Brequinar (BRQ) was given 15 mg/kg intraperitoneally (IP) every three days, dissolved in 10% DMSO and 5% Tween-80 in PBS. When imaging, 1% pentobarbital sodium was used at 80 mg/kg via intraperitoneal injection. Mice were euthanized when the tumor volume reached 2000 mm^3^. In addition to tumor volume, any mouse that showed symptoms of tumor ulceration, cachexia, dehydration, loss of body weight of 20%, inability to get food and water, or paralysis was regarded to reach a humane endpoint and would be euthanized. Mice were euthanized by carbon dioxide asphyxiation at the flow rate of 30–70% of the chamber volume per minute. Death was confirmed by continuous exposure to CO_2_ for at least 15 min after respiratory arrest.

### Histology and immunohistochemistry

Fresh tumor tissues were fixed in 10% neutral buffered formalin overnight, washed once with PBS, and stored in 70% ethanol at 4 °C. The tissues were dehydrated and embedded in paraffin according to standard protocols. Embedded tissues were sectioned at a thickness of 3 μm for H&E or immunohistochemistry analysis. IHC analysis was performed according to the antibodies protocol. The primary antibodies, including c-myc (1:200, 5605, Cell Signaling Technology), RPL26L1 (1:200, ABS146832, Absin), RPS27 (1:200, ABS138642, Absin) MRPS6(1:200, ABS118709, Absin) were incubated overnight at 4 °C. Staining was performed using the Images were randomly taken from the renal cortex (three images per tumor) at × 400 magnification using an Olympus microscope.

### Statistical analysis

The unpaired Student’s t-test was used to compare the two groups’ differences One-way ANOVA followed by Dunnett’s or Turkey’s test was used to compare the difference between more than two groups. Two-way ANOVA followed by Bonferroni post-hoc analysis was used to compare the difference between groups in two factors. The specific statistical method was described in the graph’s legend. Statistical analysis was performed using GraphPad Prism version 7.0 (GraphPad Software, Inc.). Data from three independent experiments are shown as the mean ± standard error (SE). *P* values < 0.05 were considered significant.

## Results

### Activity of DHODH inhibition in genetically defined double-hit DLBCL cell lines

**Fig. 1:**
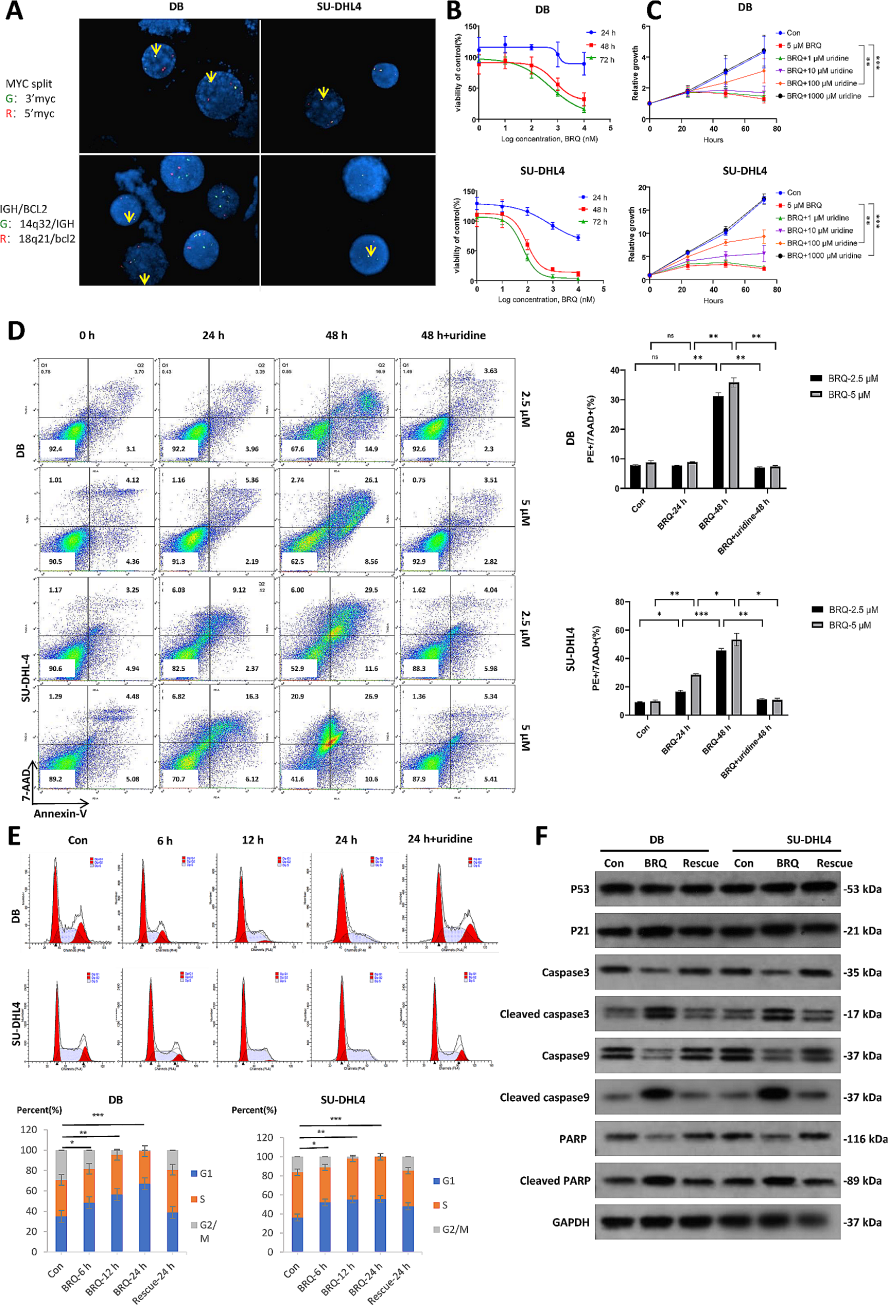
The DHODH inhibitor represses the growth of human DHL cells. (**A**) FISH analysis of DB and SU-DHL4 cells confirmed c-MYC and BCL2 translocation. Separate probes were used, and the split points were labeled by yellow arrows. (**B**) Dose-dependent effects of brequinar (BRQ) on the viabilities of DB and SU-DHL-4 cells. The cells were treated with DMSO as the control or 1 ∼ 10,000 nM BRQ for 24 to 72 h. The cell viability was calculated by the formula: Cell viability (%) = [OD (drug+) - OD (Blank)] / [OD (drug-) - OD (Blank)] × 100% at 24 h, 48 h and 72 h. Data was normalized as 100% by the formula: OD (drug-) - OD (Blank). The x-axis log 0 means 1 nM BRQ. (**C**) Cell proliferation assay of DB and SU-DHL4 cells treated with DMSO or 5 µM BRQ with or without uridine at the indicated time points. Significance was achieved by one-way ANOVA followed by Dunnett’s test. *, *P* < 0.05, **, *P* < 0.01, ***, *P* < 0.001. (**D**) Flow cytometry cell apoptosis assays were used in DB and SU-DHL4 cells with DMSO (con), 2.5 µM, or 5 µM BRQ and 5 µM BRQ plus 1000 µM uridine (rescue) for 24 and 48 h. Significance was achieved by two-way ANOVA followed by Bonferroni post-hoc analysis. *, *P* < 0.05, **, *P* < 0.01, ***, *P* < 0.001. (**E**) Cell cycle analysis in BRQ-treated DLBCL cells by flow cytometry using propidium iodide (PI). DB and SU-DHL-4 cells were treated with DMSO or 5 µM BRQ for 6, 12, and 24 h and 5 µM BRQ plus 1000 µM uridine for 24 h. The percentage of cells in G2/M phases is indicated in the histogram. ***, *P* < 0.001. (**F**) The total protein levels of P53, P21, caspase3, caspase9, and PARP and the cleaved versions of caspase proteins in DB and SU-DHL4 cells were detected by western blotting after treatment with DMSO, 5 µM BRQ, and 5 µM BRQ plus 1000 µM uridine for 48 h. Data are shown as means ± standard error (SE). All experiments were performed in triplicate

First, we confirmed the rearrangements of MYC and BCL2 in two HGBCL cell lines, DB and SU-DHL4, by FISH and WB (Fig. 1A and Figure S1B). The SU-DHL2 was chosen as a control cell line without rearrangements of MYC or BCL2, which was confirmed by FISH and WB (Figure S1A and Figure S1B). Then, we analyzed the time-dependent and dose-dependent inhibitory effects of the DHODH inhibitor brequinar (BRQ) and the FDA-approved immunomodulatory drug leflunomide(LEF) on the survival of DB, SU-DHL4 and SUDHL2 cells. HGBCL cells displayed sensitivity to brequinar (Fig. 1B), whereas SU-DHL2 was resistant to brequinar (Figure S1C). After excluding the effects of uridine on the viability of cells (Figure S2A), the effects of DHODH inhibition were rescued by supplying the cells with high concentrations of exogenous uridine (Fig. 1C). The results indicated that lymphoma cells depended on DHODH for intracellular uridine synthesis and could survive on the salvage pathway, including uridine uptake from the environment. Furthermore, DHODH inhibition induced noticeable apoptosis and G1/S phase blockade in HGBCL cells, which were rescued by uridine supplementation (Fig. 1D and E). LEF could also inhibit proliferation and induce apoptosis in DB and SU-DHL4, but the efficiency was much lower than BRQ (Figure S2B-C). For the control cell line SU-DHL2, DHODH inhibition can not induce apoptosis and G1/S phase blockade (Figure S1D-E). The effects of brequinar on apoptosis and the cell cycle were confirmed by the detection of related protein expression. The results showed that brequinar treatment upregulated p21, as well as the cleavage of caspase 3, caspase 9, and PARP, but not p53, indicating that the effects of brequinar might be p53-independent (Fig. 1F). P53 was evaluated by Sanger sequence. SUDHL4 cells carry wide type P53, but DB cells carry mut P53 (Figure S4A). No matter the wide type or the mutant type, the expression was not changed by BRQ. To further confirm the effects of brequinar by targeting DHODH, we constructed DHODH knockout (KO) cells. The results showed that DHODH knockout inhibited the proliferation of SU-DHL4 cells through cell cycle arrest and apoptosis (Figure S2D-F). These data verified that DHODH inhibition could repress HGBCL cell lines with MYC and BCL2 abnormalities.

### Inhibition of DHODH could downregulate c-MYC expression

**Fig. 2:**
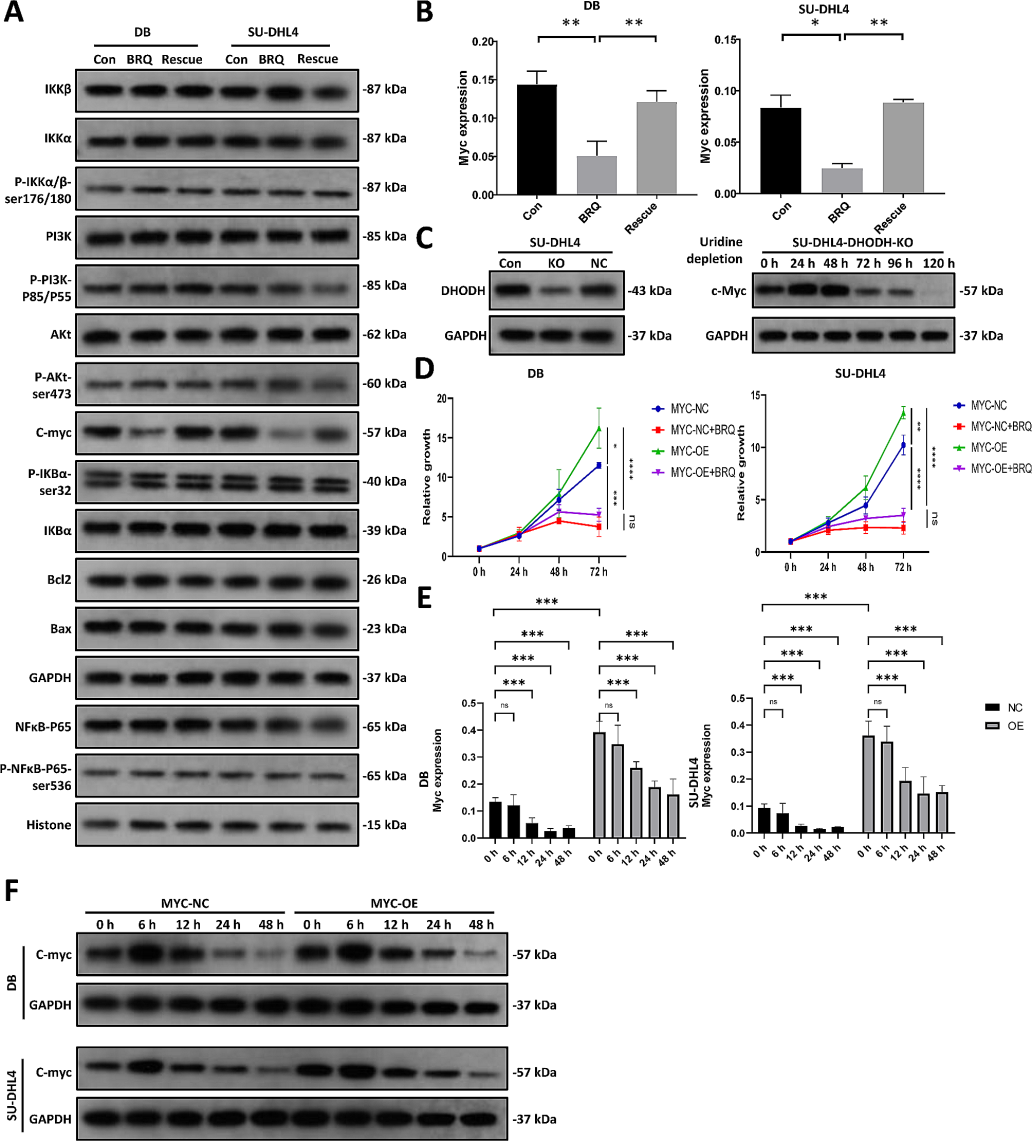
DHODH inhibition downregulates MYC expression in DHL cells. (**A**) Total protein levels of c-MYC, BCL-2, and BAX and the protein levels of the NF-κB pathway, PI3K/AKT pathway in DB and SU-DHL4 cells treated with DMSO, 5 µM BRQ, and 5 µM BRQ plus 1000 µM uridine for 48 h. Total proteins were extracted from these cells and subjected to western blotting using the indicated antibodies (left panel). (**B**) Gene expression of MYC in DB and SU-DHL4 cells incubated with DMSO, 5 µM BRQ, and 5 µM BRQ plus 1000 µM uridine for 48 h. The values are shown as the mean ± SE of 3 independent experiments. Significance was achieved by one-way ANOVA followed by Dunnett’s test. *, *P* < 0.05, **, *P* < 0.01, ***, *P* < 0.001. (**C**) The protein levels of c-MYC in DHODH knockout (KO) SU-DHL4 cells. DHODH-KO cells were cultured in medium with 1000 µM uridine to keep the cells survival. The effects of uridine withdrawal on the c-MYC expression were examined at indicated time points after culturing cells in medium without uridine. (**D**) Cell proliferation assay of DB and SU-DHL4 cells overexpressing MYC (MYC-OE) or empty vector (NC). The cells were treated with DMSO or 5 µM BRQ at the indicated time points. NC, empty vector. The values are shown as the mean ± SE of 3 independent experiments. Significance was achieved by one-way ANOVA followed by Turkey’s test. *, *P* < 0.05, **, *P* < 0.01, ***, *P* < 0.001. (**E**) Gene expression of MYC in MYC-OE or MYC-NC cells incubated with 5 µM BRQ at the indicated time points. The values are shown as the mean ± SE of 9 independent experiments. Significance was achieved by two-way ANOVA followed by Bonferroni post-hoc analysis. *, *P* < 0.05, **, *P* < 0.01, ***, *P* < 0.001. (**F**) The protein level of c-MYC in MYC-OE or MYC-NC cells treated with 5 µM BRQ

We then confirmed that c-MYC could be repressed by brequinar and LEF at the protein and mRNA levels in HGBCL cells (Fig. 2A-B and Figure S3A). The effects were rescued by adding uridine to the medium. BCL2 and its partner BAX were not affected by brequinar (Fig. 2A). Consistently, the c-MYC gene levels in DHODH-knockdown cells decreased gradually after uridine withdrawal (Figure S2G). Notably, the protein level of c-MYC was upregulated temporarily after uridine absence and then downregulated by continuous uridine depletion (Fig. 2C). Overexpression of c-MYC promoted the proliferation of HGBCL cells; however, the effects were blocked by brequinar treatment (Fig. 2D). Even the MYC level in the MYC OE cells was downregulated by brequinar at the mRNA and protein levels (Fig. 2E and F). The inhibition effect of LEF on MYC expression is weaker than BRQ. Under the treatment of LEF, MYC was downregulated at 48 h (Figure S3A), while MYC was downregulated at around 12 h after BRQ treatment. Consistent with uridine withdrawal, the protein level of c-MYC was upregulated 6 h after brequinar treatment and then downregulated gradually with prolonged treatment time. The gene levels of c-MYC decreased over time. These data indicated that the expression of c-MYC was strictly regulated by intracellular uridine.

The PI3K/AKT and NFκB pathways are highly active in many B cell malignancies and have regulatory effects on c-MYC in terms of stability and expression [18, 19]. To investigate the potential upstream regulators of c-MYC on brequinar treatment, we evaluated the protein expression levels of the PI3K/AKT and NFκB pathways. We found that brequinar did not affect specific proteins, including PI3K, AKT, IKKα, IKKβ, IKBβ, and p65 (Fig. 2A). Previous findings have demonstrated that the JAK/STAT pathway regulates MYC gene expression [20]. Therefore, we also detected alterations in the JAK/STAT pathway upon brequinar treatment. However, no obvious findings were observed in brequinar-treated cells compared with controls (Figure S3B), indicating that other regulatory mechanisms require further study.

### Effect of combined targeting of DHODH and BCL2 in DHL cells

**Fig. 3:**
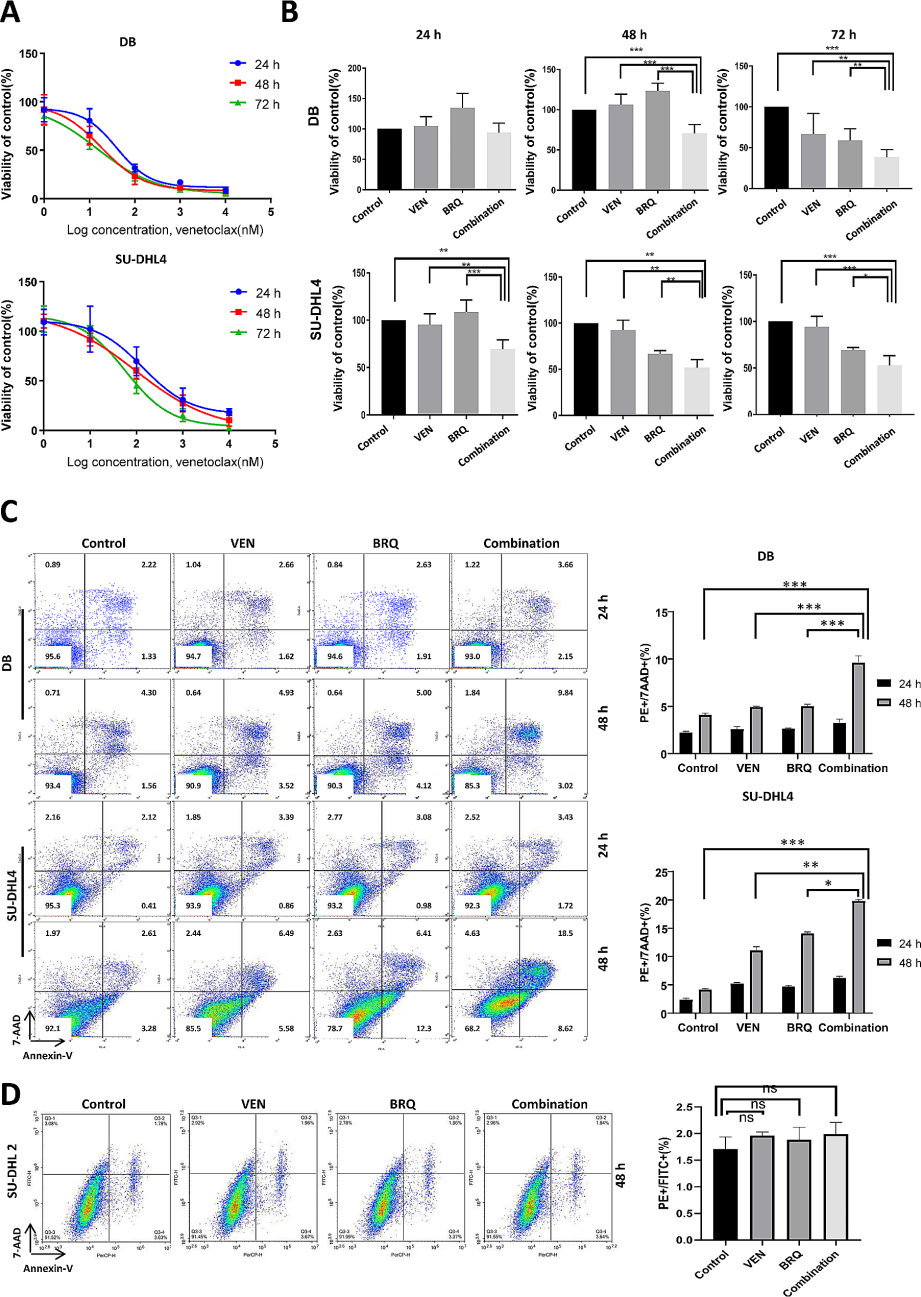
The combination of BRQ with venetoclax synergistically inhibits the growth of DHL cells. (**A**) Cell viability of DB and SU-DHL4 cells treated with the indicated concentrations of venetoclax for 24, 48, and 72 h. (**B**) Cell viability of DB and SU-DHL4 cells treated with DMSO (Control) or 20 nM venetoclax (VEN), 500 nM BRQ, or 20 nM venetoclax plus 500 nM BRQ (Combination) for 24, 48, and 72 h. The values are presented as the mean ± SE of 3 independent experiments. Significance was achieved by one-way ANOVA followed by Dunnett’s test. ***, *P* < 0.001, **, *P* < 0.01, *, *P* < 0.05. (**C**) Apoptosis assay of DB and SU-DHL4 cells treated with DMSO (Control) or 20 nM venetoclax (VEN), 500 nM BRQ, or 20 nM venetoclax plus 500 nM BRQ (Combination) for 24 and 48 h. Experiments were repeated three times. Significance was achieved by one-way ANOVA followed by Dunnett’s test. ***, *P* < 0.001, **, *P* < 0.01, *, *P* < 0.05. (**D**) Apoptosis assay of SU-DHL2 cells treated with DMSO (Control) or 20 nM venetoclax (VEN), 500 nM BRQ, or 20 nM venetoclax plus 500 nM BRQ (Combination) for 48 h. Experiments were repeated three times. Significance was achieved by one-way ANOVA followed by Dunnett’s test. ***, *P* < 0.001, **, *P* < 0.01, *, *P* < 0.05

Since HGBCL likely depends on both MYC and BCL2 with distinct pathways, targeting both oncogenes is a rational therapeutic approach. First, we confirmed that venetoclax (VEN) could inhibit the viability of DB and SU-DHL4 cells (Fig. 3A). To evaluate the possible synergism, we treated DB and SU-DHL4 cells with increasing concentrations of brequinar and venetoclax as single agents or in combination. These two agents exerted an enhanced effect on the survival of HGBCL cell lines. The doses of 500 nM brequinar and 20 nM venetoclax 48 h after treatment had the most apparent synergistic effects on both cell lines (Fig. 3B and Supplementary Table 2). Apoptotic cells were increased by the combination of brequinar with venetoclax in HGBCL cells, but not in control cells (Fig. 3C-D). However, these two reagents had no obvious synergistic effects on the cell cycle in HGBCL cells and control cells (Figure S4B).

**Fig. 4:**
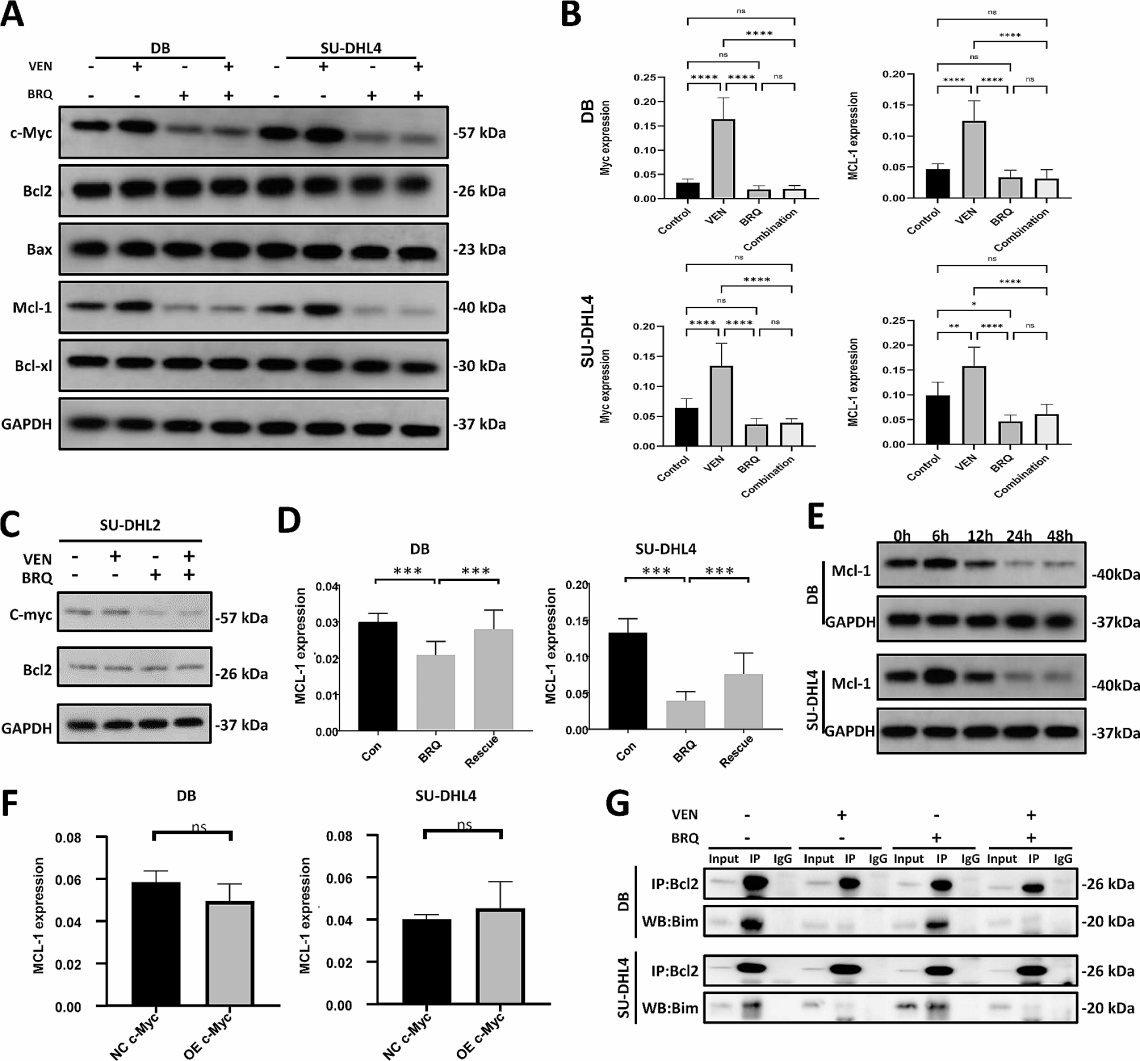
The combination of brequinar and venetoclax affects DHL cells through complementary pathways. (**A**) The protein levels of c-MYC, BCL-2, BAX, Mcl-1, and BCL-XL in DB and SU-DHL4 cells treated with DMSO (control), 20 nM venetoclax (VEN), 500 nM BRQ, and 20 nM venetoclax plus 500 nM BRQ (combination) for 48 h. (**B**) Gene expression of c-MYC and MCL-1 from DB and SU-DHL4 cells treated with single or combined venetoclax and BRQ for 48 h. The values were calculated from 6 independent experiments. Significance was achieved by one-way ANOVA followed by Turkey’s test.****, *P* < 0.0001, ***, *P* < 0.001, **, *P* < 0.01, *, *P* < 0.05. (**C**) The protein levels of c-MYC and BCL-2 in SU-DHL2 cells treated with DMSO (control), 20 nM venetoclax (VEN), 500 nM BRQ, and 20 nM venetoclax plus 500 nM BRQ (combination) for 48 h. (**D**) Gene expression of MCL-1 downregulated by BRQ could be rescued by uridine. DB and SU-DHL4 cells were DMSO, 5 µM BRQ, and 5 µM BRQ plus 1000 µM uridine for 48 h. The values are shown as the mean ± SE of 3 independent experiments. Significance was achieved by one-way ANOVA followed by Dunnett’s test. **, *P* < 0.01, ***, *P* < 0.001. (**E**) The protein level of MCL-1 in DB and SU-DHL4 cells treated with DMSO or 5 µM BRQ at the indicated time points (hours). (**F**) Gene expression of MCL-1 in MYC-NC and MYC-OE DB and SU-DHL4 cells. The values are shown as the mean ± SE of 3 independent experiments. (**G**) Protein-protein interactions of BCL-2/BIM in SU-DHL4 and DB cells. The cells were treated with DMSO, 20 nM venetoclax, 500 nM BRQ, and 20 nM venetoclax plus 500 nM BRQ for 48 hours

Venetoclax binds to BCL2 and releases proapoptotic proteins, including BIM, causing BAX-mediated apoptosis [21]. We then examined the protein levels of c-MYC, BCL2, BAX, and MCL-1 in HGBCL cell lines after brequinar and/or venetoclax treatment. We found that venetoclax treatment upregulated both MCL-1 and c-MYC, which have been reported to be partially responsible for venetoclax resistance [22]. Brequinar showed the opposite effects of decreasing both the mRNA and protein levels of MCL-1 and c-MYC (Fig. 4A and B). To dismiss the possibility that the treatments were merely killing cells with lower levels of the proteins, we treated the cells with caspase inhibitors and observed similar results (Figure S5A-B). For the control cell line, venetoclax treatment could not upregulate c-MYC, but Brequinar showed a downregulation effect on c-MYC, though the effect is weak (Fig. 4C). MYC has been reported to regulate the gene expression of MCL-1 in some solid tumors [23]. Here, the expression levels of MCL-1 were downregulated gradually upon brequinar treatment, which could be rescued by uridine (Fig. 4D and E). The alterations of MCL-1 were not synchronous with c-MYC. Overexpression of c-MYC did not increase the gene expression of MCL-1 (Fig. 4F). These data indicated that MCL-1 was regulated upon uridine depletion in a route that was likely independent of c-MYC, and thus the molecular mechanism merits further investigation. Additionally, venetoclax could displace BIM from BCL2 in DB and SU-DHL4 cells, as previously reported, while brequinar had no effects on the interaction between BCL2 and BIM (Fig. 4G).

**Fig. 5:**
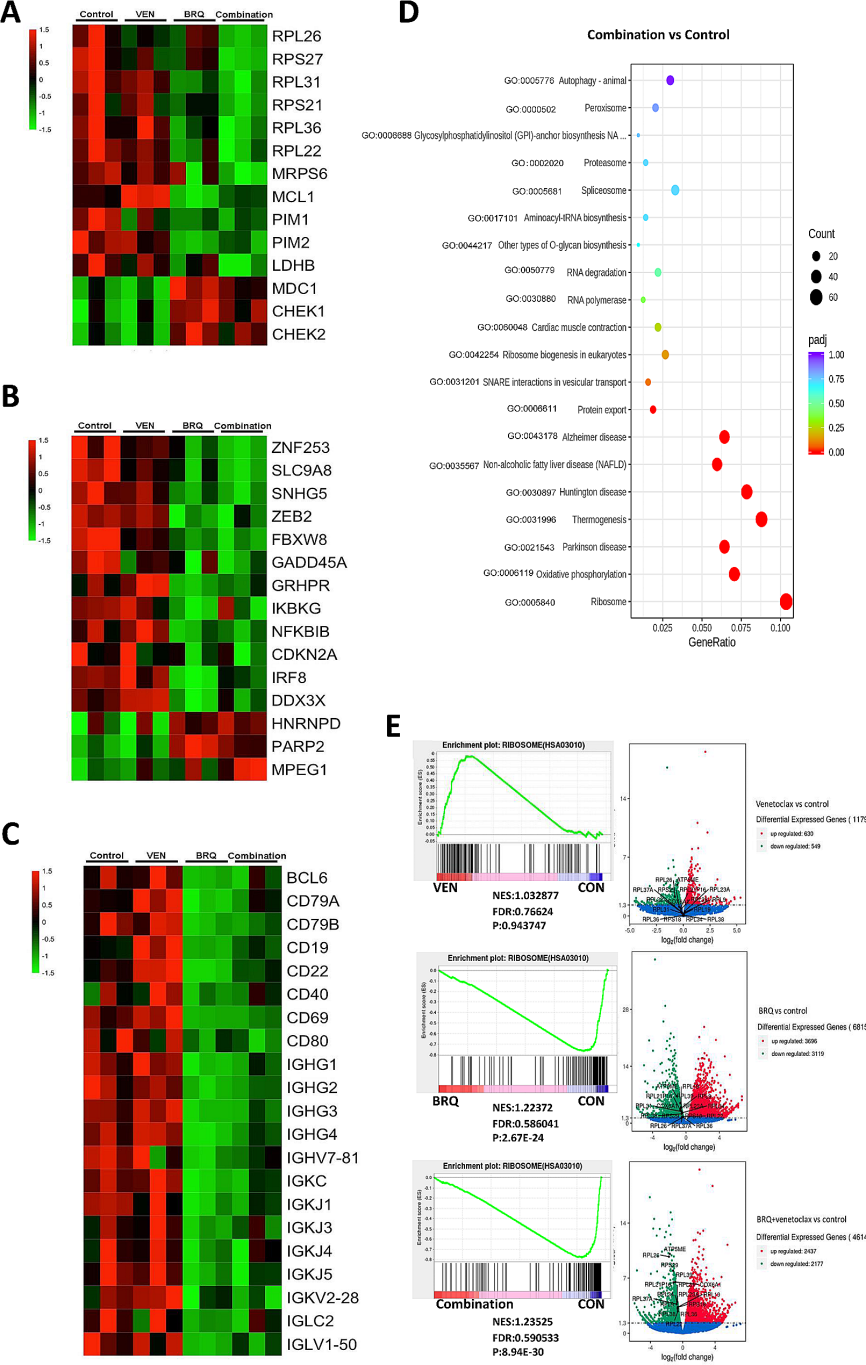
Venetoclax and BRQ treatments lead to distinct gene expression profiles. (**A**) Differentially expressed genes involved in ribosome biosynthesis, DNA repair, and damage pathway in SU-DHL4 cells after treatment with venetoclax and/or BRQ for 48 h. (**B**) Genes reported to be mutated in DLBCL cells differentially expressed in SU-DHL4 cells. (**C**) Genes regulating the function of mature B cells differentially expressed in SU-DHL4 cells. (**A-C**) Significantly dysregulated genes are represented in the heatmap. The color scale bar represents higher (red) to lower (green) expression. Gene expression levels are expressed as FPKM values, and differences are shown on a color scale after Z-score transformation. FPKM, fragments per kilobase of exon per million fragments mapped. (**D**) GO enrichment analysis of SU-DHL4 cells treated with the BRQ and venetoclax combination compared with DMSO (control). (**E**) Gene set enrichment analysis enrichment plot of ribosome biosynthesis genes from data obtained 48 h after stimulation as indicated. VEN, venetoclax; BRQ, brequinar; CON, Control

**Fig. 6:**
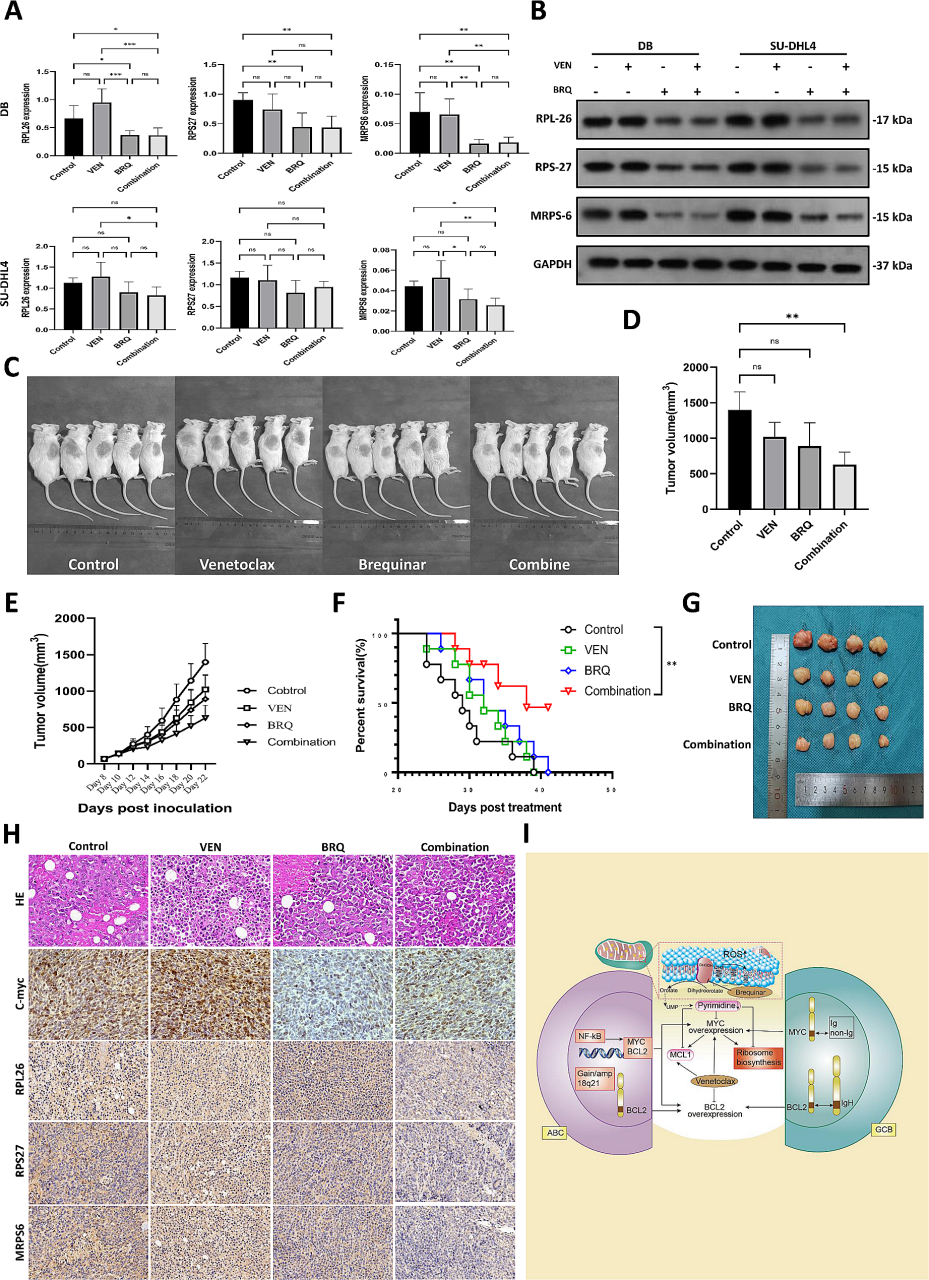
BRQ downregulates ribosome biosynthesis genes and the combination effect in DHL xenografts. (**A**) Gene expression of RPL26, RPS27, and MRPS6 in SU-DHL4 cells incubated with DMSO (Control) or 20 nM venetoclax (VEN), 500 nM BRQ, or 20 nM venetoclax plus 500 nM BRQ (Combination) for 48 h. The values are shown as the mean ± SE of 6 independent experiments. Significance was achieved by one-way ANOVA followed by Turkey’s test. *, *P* < 0.05, **, *P* < 0.01, ***, *P* < 0.001. (**B**) Protein levels of RPL-26, RPS-27 and MRPS-6 from DB and SU-DHL4 cells treated with DMSO, 20 nM venetoclax, 500 nM BRQ, and 20 nM venetoclax plus 500 nM BRQ for 48 h. (**C**) Images of the tumor-bearing NOD-SCID mice treated with vehicles(control), venetoclax, brequinar, venetoclax and brequinar combination at 20 days after inoculation. (**D**) Comparison of tumor volumes in tumor-bearing NOD-SCID mice treated with venetoclax, brequinar, or venetoclax and brequinar combination to vehicles(control) at the end of treatment. The values are presented as the mean ± SD (*n* = 4). Significance was achieved by one-way ANOVA followed by Dunnett’s test. ***, *P* < 0.001, **, *P* < 0.01, *, *P* < 0.05, ns, non-significance. (**E**) Tumor growth curves in tumor-bearing NOD-SCID mice after SU-DHL4 cells inoculation. (**F**) Survival analysis of the DHL xenograft mice was performed using a log-rank test for comparison. (**G**) Stripped tumor tissue images from xenograft tumors with the indicated treatments at day22 post incubation. (**H**) Representative HE and immunochemical images from xenograft tumors with the indicated treatments at day 22 post incubation. (**I**) A schematic diagram showing the synergistic mechanism of DHODH and BCL-2 inhibitors

DB and SU-DHL4 cells have been reported to be venetoclax-sensitive cells [24]. To further verify the effect of combining the two drugs, we chose SU-DHL10 as venetoclax-resistant cells that showed no response to venetoclax treatment (Figure S6A). SU-DHL10 cells were susceptible to brequinar, and uridine supplementation rescued these effects (Figure S6B). Like DB and SU-DHL4 cells, c-MYC and MCL-1 in SU-DHL10 cells were downregulated upon brequinar treatment (Figure S6C-D). Moreover, the combination of venetoclax and brequinar showed no synergistic effect on the viability of SU-DHL10 cells, probably because the cells lacked BCL-2 (Figure S6D-E). Taken together, the synergistic mechanism of brequinar and venetoclax is the complementarity of different pathways.

### The combination of brequinar and venetoclax leads to distinct gene expression profiles

To understand the genome-wide effects and target genes of brequinar and the combination with venetoclax, we performed an RNA-seq of SU-DHL4 cells treated with brequinar, venetoclax, or the combination of both agents versus DMSO-treated cells (blank control).

Venn analysis revealed a broader set of genes differentially expressed in cells treated with combined venetoclax and brequinar compared with the blank control or either of the compounds (*P* value < 0.05) (Figure S7A). Among these genes, some were reported to be involved in DNA damage or mutated in HGBCL cells (Fig. 5A and B). Furthermore, multiple genes regulating the function of mature B cells were dysregulated in the cotreatment group (Fig. 5C). These data indicated that the combination of venetoclax and brequinar could have synergistic effects through several pathways. GO pathway enrichment analysis confirmed that ribosome pathway genes (GO:0005840) were the most significantly associated with brequinar in SU-DHL4 cells (Fig. 5D, Figure S7B-C). KEGG enrichment analysis further identified that ribosome pathway gene (hsa03010) sets were negatively enriched in both brequinar- and combination-treated cells (Fig. 5E and Figure S7D). Consistent with the RNA-seq data, the transcriptional and protein levels of several genes involved in ribosome pathways, including RPL26, RPS27, and MRPS6, were significantly decreased after brequinar or combination treatment (Fig. 6A and B). In SU-DHL4 cells, we observed the trend of downregulation of ribosome proteins (Eventhough not statistically significant). The effects of brequinar on the expression levels of ribosome pathway genes could be repressed by brequinar and rescued by uridine supplementation (Figure S8A-C). Ribosome biogenesis is a finely regulated multistep process, and one of the master regulators of ribosome biogenesis is MYC [25]. Here, overexpression of c-MYC showed no obvious effects on the expression of RPL26, RPS27, and MRPS6 (Figure S8D). The relationship between c-MYC and ribosome biogenesis in DHL cells upon brequinar treatment requires a deeper evaluation.

### Venetoclax and brequinar Combination demonstrates effective tumor control in DHL xenografts

To further validate the in vivo anti-tumor effect of venetoclax and brequinar combination, we constructed the DHL xenograft mice model by injecting SU-DHL4 cells into NOD-SCID mice. The tumor-bearing mice were then divided randomly to receive vehicle control, venetoclax (50 mg/kg once daily by oral gavage for a total of 14 days) / brequinar (15 mg/kg intraperitoneally every three days for a total of 6 does) monotherapy, or a combination of venetoclax and brequinar(Fig. 6C). The dose of venetoclax and BRQ was chosen based on previously published literature [16, 26]. Both venetoclax and brequinar monotherapy failed to demonstrate any therapeutic effect compared to the vehicle controls in these models. The brequinar monotherapy seemed more effective than venetoclax, but the difference was not statistically significant. However, the combination treatment of venetoclax and brequinar elicited tumor growth delays compared with the vehicle control and monotherapy group. At the end of treatment, tumor volumes in mice receiving both venetoclax and brequinar showed a significant reduction compared to those in the control group (Fig. 6D and E). Furthermore, these tumor control effects increased overall survival (Fig. 6F) without increasing more toxicity(Figure S8E).

Moreover, consistent with our in vitro results, immunohistochemical staining of fresh tumor samples showed that the expression of MYC and ribosome biogenesis proteins were downregulated by BRQ monotherapy or combination with venetoclax (Fig. 6G-H, Figure S8F). Our in vivo study showed that the combination of DHODH and BCL2 blockade is a potential new strategy for DLBCL with abnormal MYC and BCL2.

## Discussion

It has been recognized that patients with HGBCL lymphoma have inferior prognoses and are less responsive to standard treatment. Clinical efforts to improve outcomes for these patients have largely involved intensifying, modifying, or replacing the CHOP backbone and adding new drugs to R-CHOP [27, 28]. Although such alternatives may be offered by particular populations, none of those trials have shown a statistically significant improvement in the cure rate and progression-free survival. Thus, a comprehensive understanding of the molecular mechanism and identifying the key regulators as potential targets are underway. Combination therapy targeting both MYC and BCL2 may provide a new strategy to improve the clinical outcome of MYC*/*BCL2 double-hit lymphomas and MYC/BCL2 dual expressers.

As a powerful transcription factor, MYC plays a vital role in tumor pathogenesis and development, with a wide range of biological activities, including apoptosis, growth, proliferation, differentiation, migration, and cellular metabolism [29, 30]. Although directly targeting MYC has been explored for several decades, until now, no effective drugs have been produced. Brequinar, a DHODH inhibitor, exerts potent differentiation activity in vitro and in vivo in both murine and human models of AML [26, 31]. A phase 1b/2a study of brequinar is ongoing for relapsed and refractory AML patients. Here, we showed that brequinar had an inhibitory effect on the survival of HGBCL cell lines by inducing apoptosis and cell cycle arrest. We found that brequinar could repress c-MYC gene expression at the transcriptional level. The results were similar to findings reported in Burkitt lymphoma [32]. However, the reported common pathways involved in the regulation of c-MYC, such as the PI3K/AKT, NFκB, and JAK/STAT pathways, were not affected by brequinar. Intriguingly, the protein level of c-MYC was upregulated temporarily after short-term brequinar exposure or uridine depletion. Both mRNA and protein levels of c-MYC were downregulated by prolonged treatment with brequinar. The effects of brequinar on HGBCL cells could be rescued by exogenous uridine supplementation. The rescue concentration of uridine was more than 100 µM, much higher than the concentration of plasma uridine [33], indicating that brequinar might have effects in vivo, which requires further study. We also found that c-MYC protein was elevated 6 h after brequinar treatment. However, the expression level of c-MYC was downregulated at the transcriptional level after sustained uridine depletion. Based on these results, we presume that c-MYC is a sensor of intracellular uridine in lymphoma cells, which can be upregulated at the protein level upon treatment with lower concentrations of uridine at earlier stages. While the transcription of c-MYC requires uridine to synthesize RNA, the mRNA levels of c-MYC decrease gradually with prolonged depletion of uridine. However, these hypotheses need further verification.

BCL2 and its family proteins function as inhibitors and activators of the intrinsic apoptotic pathway to govern the fate of cancer cells [34]. The selective BCL2 inhibitor venetoclax is a promising therapeutic strategy for cancers; however, its clinical efficacy in DLBCL is far from satisfactory. One possible reason for this limitation is that the apoptotic sensitivity to venetoclax is influenced not only by total amounts of BCL2 but also by its phosphorylation status and the additional presence of other pro-survival proteins [22, 35]. Among the pro-survival proteins, MCL-1 is considered the major determinant of resistance to venetoclax. Thus, combinations with other regimens are currently being explored. However, the survival improvement is still uncertain, and higher grade 3–4 adverse events (AEs, 85%) need to be considered [36, 37]. Reduction of the venetoclax dosage may help to alleviate the toxicity. For DHL or DEL, both BCL2 and MYC play roles in pathogenesis. Therefore, a combined approach involving MYC, BCL2, and even MCL-1 may be promising to overcome the poor response of HGBCL.

In this study, the BCL2 inhibitor venetoclax could repress lymphoma growth by interfering with the interaction between BCL2 and BIM. However, lymphoma cells could upregulate MCL-1 and MYC upon venetoclax treatment, which might cause resistance to venetoclax. Treatment with the DHODH inhibitor brequinar downregulated MCL-1 and MYC, possibly enhancing the effect of venetoclax in HGBCL cells. The different effects of the two drugs could reasonably support synergy, avoiding resistance in HGBCL cells. As shown in our in vivo experiments, venetoclax and brequinar monotherapy failed to demonstrate a noticeable therapeutic effect. In comparison, the combination treatment of venetoclax and brequinar elicited significant tumor growth delays. However, more experiments should be conducted to optimize the synergistic dosage and enhance anti-tumor ability in the future.

It has recently been reported that overexpression of MYC and BCL2 predicts a poor prognosis in patients with extranodal NK/T-cell lymphoma (NKTL) of the nasal type [38]. Theoretically, combination with brequinar could enhance the effects of venetoclax in NKTL cells with high expression of both MYC and BCL2, which is worth further investigation. MYC directs the transcription of MCL-1 directly in gastric cancer cells [23]; however, we did not observe a transcriptional regulation of MCL-1 by MYC. Therefore, we presumed that MCL-1 and MYC work cooperatively in HGBCL, as reported in a previous study on breast cancer [39]. Intriguingly, brequinar showed an obvious inhibitory effect on SU-DHL10 cells resistant to venetoclax, which have very low levels of BCL-2. In contrast, brequinar showed no inhibitory effect on SU-DHL2 cells, which carried low c-MYC and BCL-2 levels. These data indicated that the effect of brequinar is related to the expression of c-MYC and might have a broad effect on DLBCL cells. Brequinar could enhance the effect of venetoclax in specific HGBCL cells with MYC and BCL abnormalities. According to our findings and previous studies, we propose the possible mechanisms involved in the synergistic effects of brequinar and venetoclax in HGBCL cells with MYC and BCL2 dysregulation (Fig. 6I). HGBCL lymphomas carry abnormal MYC and BCL2 via different mechanisms.

The effects of brequinar could be rescued by adding uridine, indicating that uridine depletion is a key regulatory point. Uridine metabolism is a complex enzymatic network and is important for multiple cellular functions, including nucleoside synthesis and homeostasis of glucose, lipids, and amino acids [40]. Through RNA-seq analysis, we found that brequinar could affect a broad range of genes at the transcriptional level, including genes involved in the DNA damage repair signaling pathway (PIM1, PIM2, MDC1, CHEK1, and CHEK2) and those mutated in DLBCL patients (SNHG5, FBXW8, CDKN2A, etc.) [41–43]. We found that brequinar could decrease a series of genes that regulate the development and function of mature B cells or lymphoma cells, including surface molecules and different regions of immunoglobulin chains. Consistently, it was reported that another DHODH inhibitor, leflunomide, could inhibit B cell antibody production by directly acting on B cells [44]. These data suggest that brequinar probably inhibits HGBCL cells by suppressing B cell functions, mimicking immunotherapy (such as rituximab, dacetuzumab, or chimeric antigen receptor CAR-T cells). Venetoclax had relatively weaker effects on these genes. Since venetoclax acts as an anti-tumor agent by competitively inhibiting the interaction between BCL-2 and proapoptotic proteins, it is very likely that this molecule affects lymphoma cells by regulating protein-protein interactions or protein functions, but not at the transcriptional level. Notably, we found that a series of ribosome pathway genes were negatively enriched in brequinar-treated cells and could be regulated by intracellular uridine. We presume that uridine metabolism is related to the ribosome biosynthesis pathway, but the accurate molecular mechanism requires further investigation. The ribosome is one of the oldest molecular machines in extant life, and very recent findings have indicated the interconnection between ribosome biogenesis and cancer [45]. Abnormal changes in ribosomes may drive cancer pathogenesis, and MYC is a master regulator [25, 46]. Knockdown of c-MYC can inhibit the transcription of ribosomal proteins, presenting opportunities for designing new targeted cancer treatments [47]. Here, we observed that overexpression of c-MYC had no obvious effects on the gene expression of RPL26, RPS27, and MRPS6. Additional evidence is needed to clarify the relationship between c-MYC and the ribosome pathway.

## Conclusions

Our study demonstrates that a combined blockade of DHODH and BCL2 could synergistically inhibit HGBCL with concurrent MYC and BCL2 abnormalities through multiple pathways. Thus, it can be reasonably expected that a clinical-grade DHODH inhibitor combined with venetoclax might improve outcomes for HGBCL patients, as well as other cancers with high expression of MYC and BCL2 or MYC and MCL-1. Our findings provide new insights into the molecular basis of this effect and offer opportunities to design new targeted treatments for lymphoma.

### Electronic supplementary material

Below is the link to the electronic supplementary material.

Supplementary Material 1

Supplementary Material 2

Supplementary Material 3

Supplementary Material 4

## Data Availability

The sequencing data in this study were deposited to Genbank (https://www.ncbi.nlm.nih.gov/bioproject/761000), the accession number is SUB10274296, PRJNA761000, and SAMN21240300. The datasets used and/or analyzed during the current study are available from the corresponding author on reasonable request.

## Abbreviations

HGBCL: High-grade B-cell lymphoma
DHODH: Dihydroorotate dehydrogenase
BCL2: B cell lymphoma 2
MCL-1: Myeloid cell leukemia-1
DLBCL: Diffuse large B-cell lymphoma
DHL: Double-hit lymphoma
DEL: Double-expressor lymphoma
CLL: Chronic lymphocytic leukemia
AML: Acute myeloid leukemia
ORR: Overall response rate
CR: Complete response
BRQ: Brequinar
VEN: Venetoclax
UMP: Uridine monophosphate
ATCC: American Type Cell Collection
KEGG: Kyoto Encyclopedia of Genes and Genomes
GO: Gene ontology
IC50: Half maximal inhibitory concentration
CI: Combination index
RPs: Ribosomal proteins
