# Supplementary figures and images for: Targeted inhibition of DHODH is synergistic with BCL2 blockade in HGBCL with concurrent MYC and BCL2 rearrangement
Source: BMC Cancer. 2024 Jun 25;24:761. doi: 10.1186/s12885-024-12534-w (PMC11197201; doi:10.1186/s12885-024-12534-w)

FIGS1


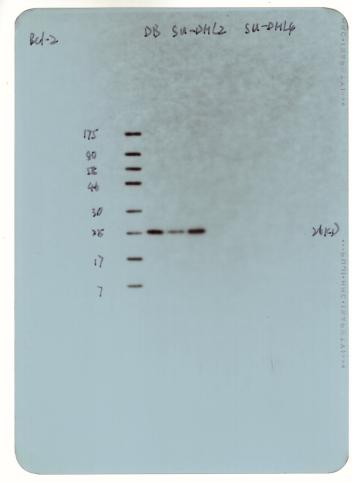

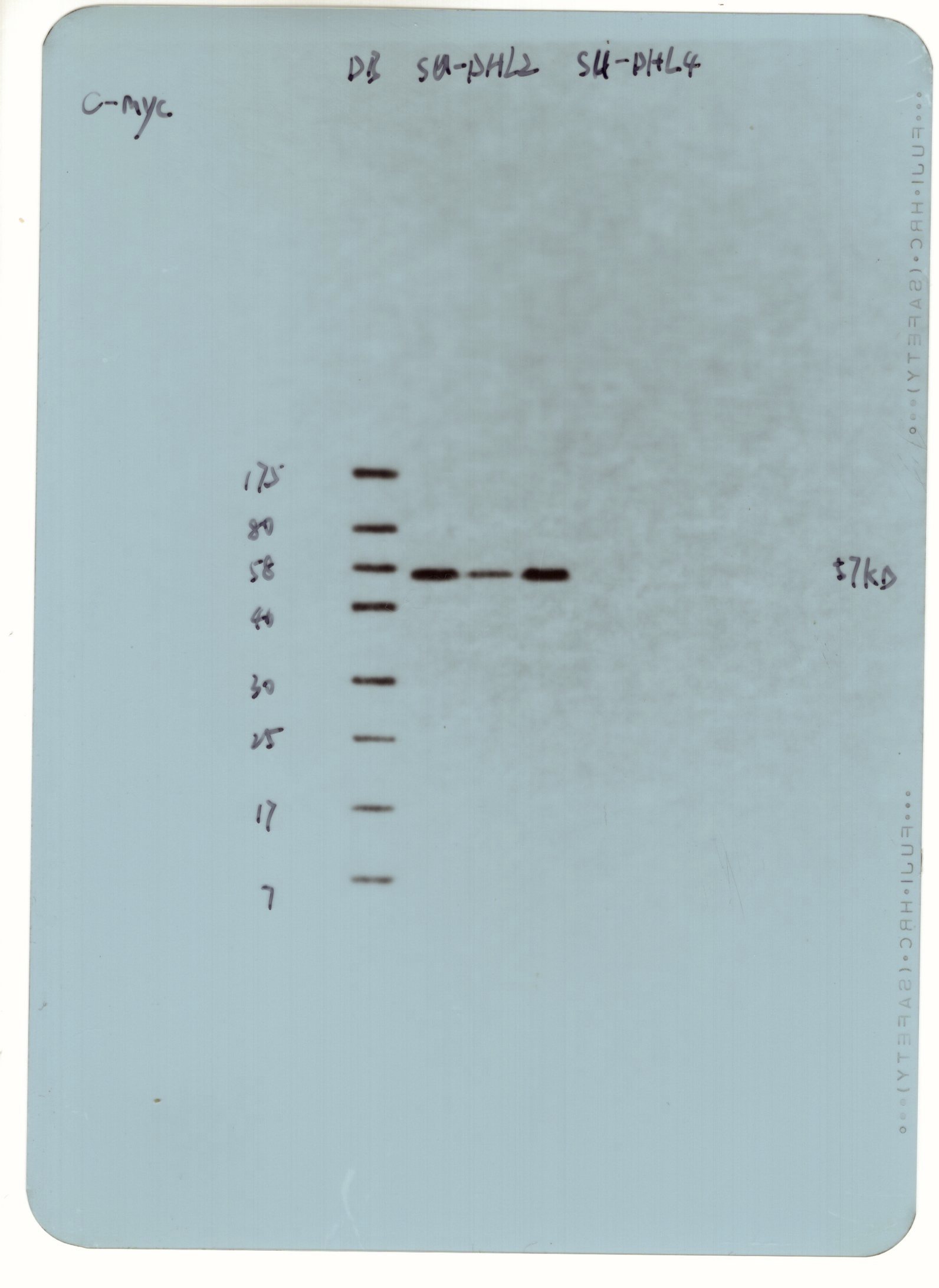

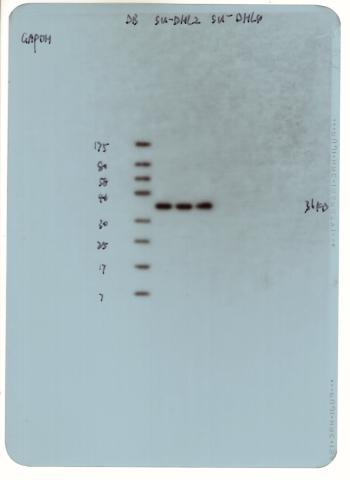


FIGS3


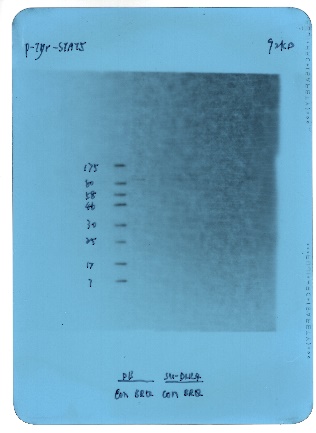

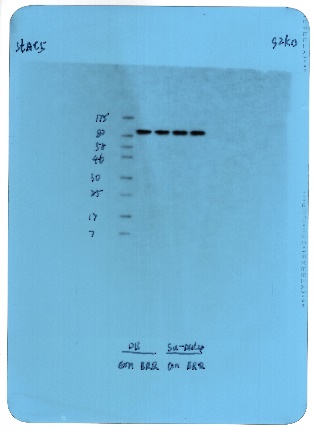

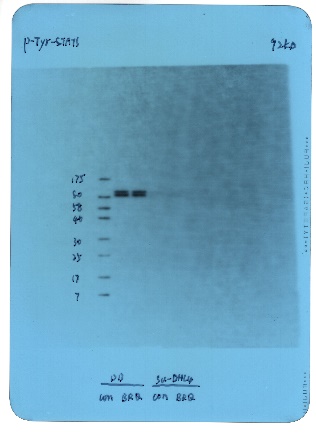

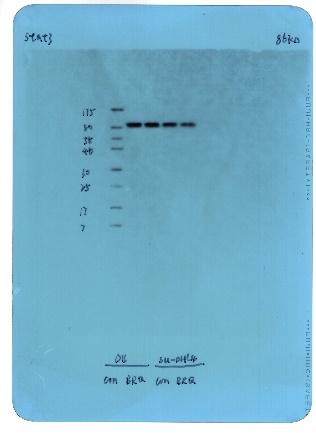

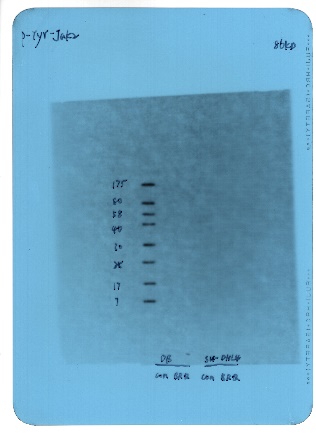

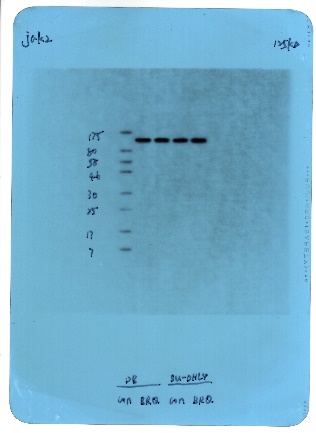

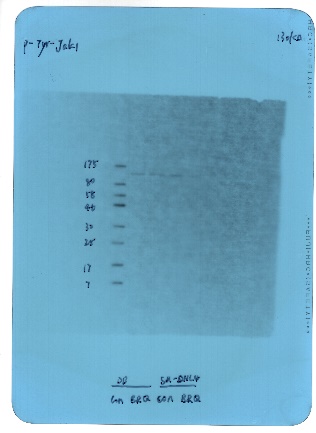

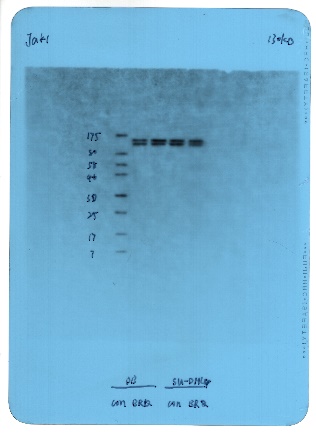

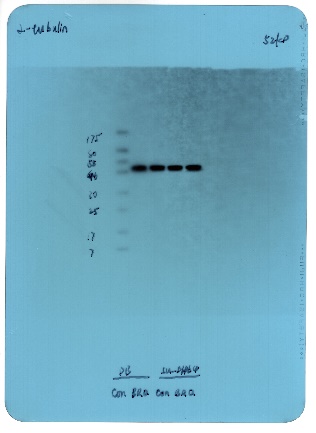


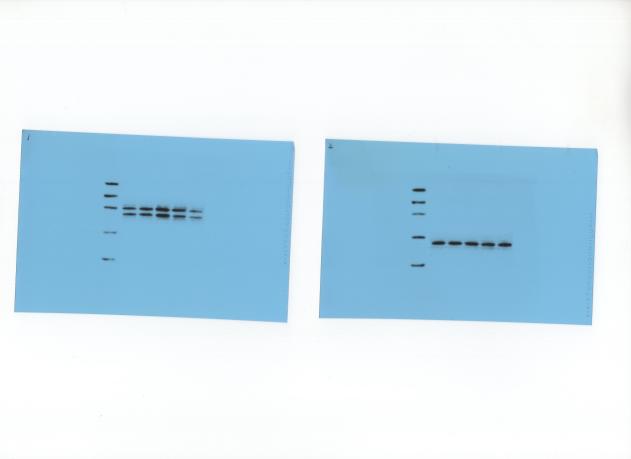

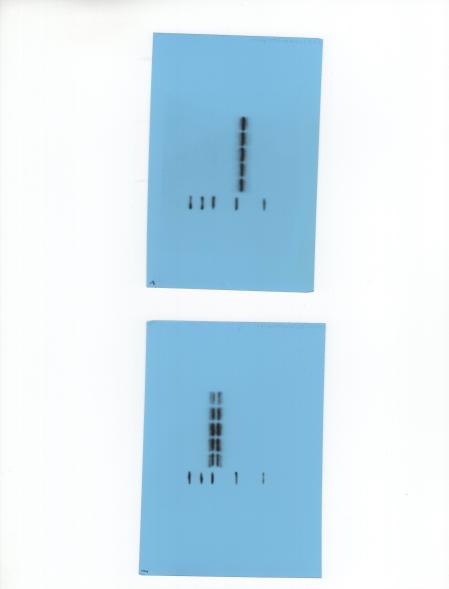


FIGS5


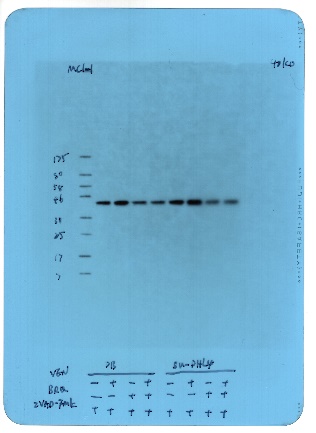

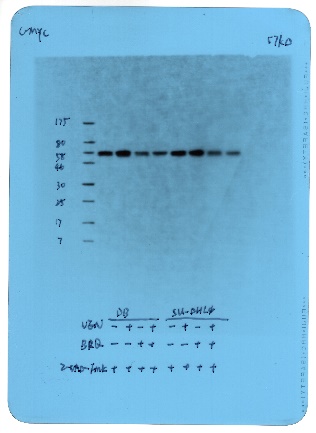

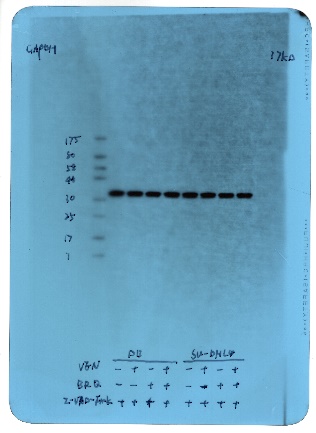


FIGS6


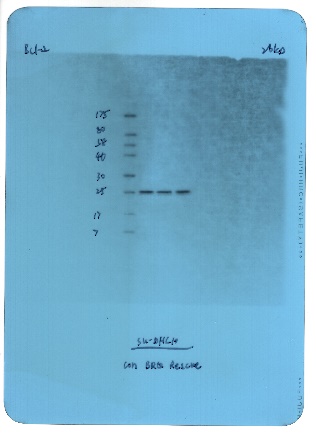

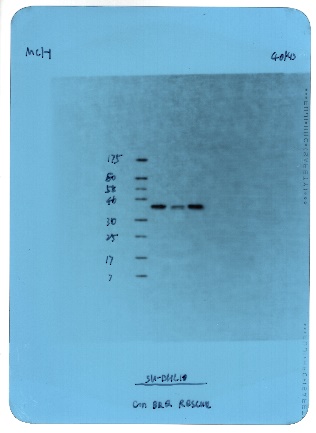

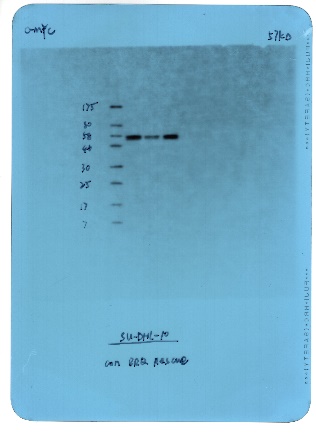

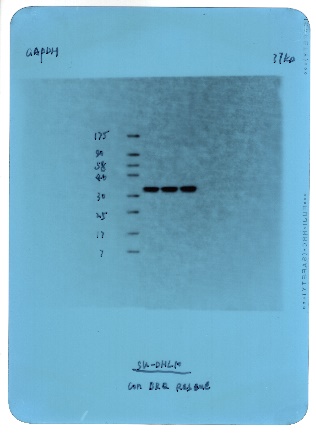


FIGS8


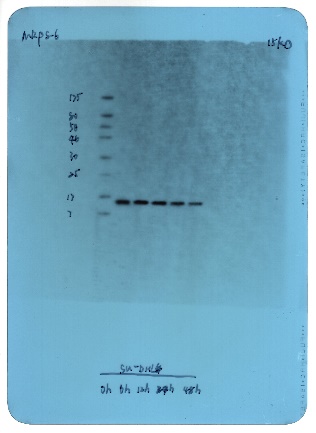

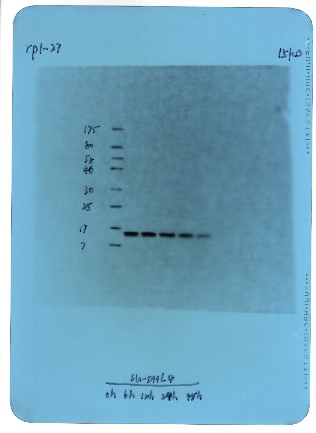

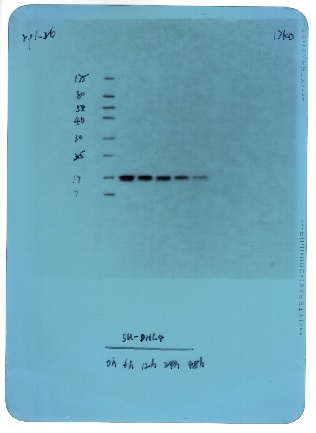

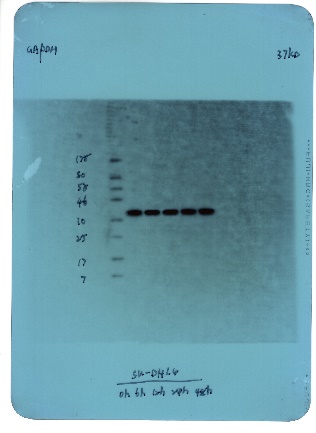

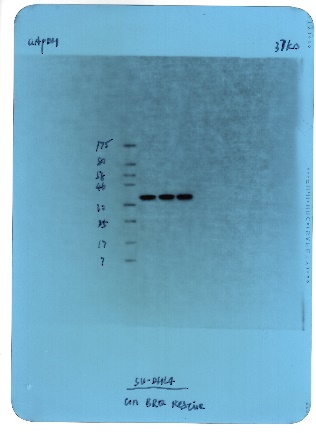

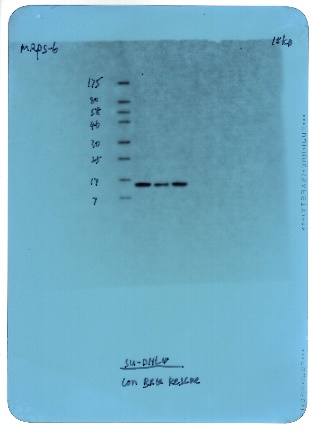

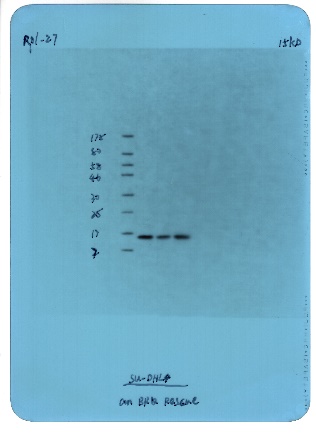

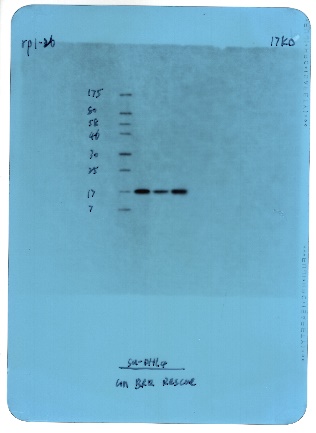

Supplement: Supplementary file 1 — Supplementary Material 1 [file 12885_2024_12534_MOESM1_ESM.docx]

FIG1


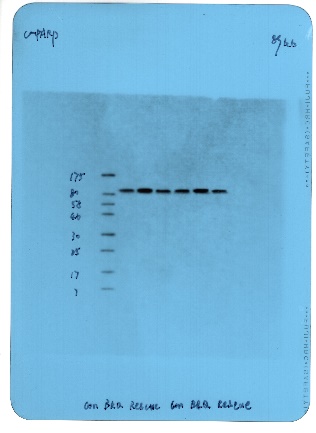

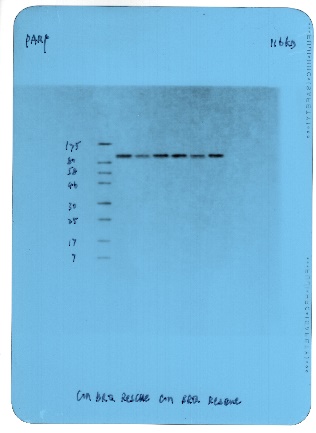

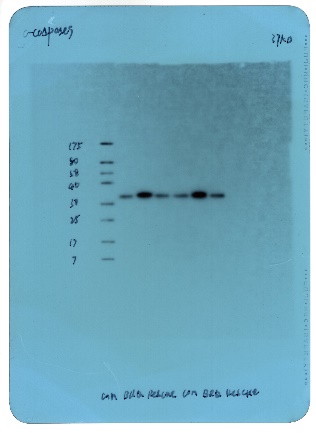

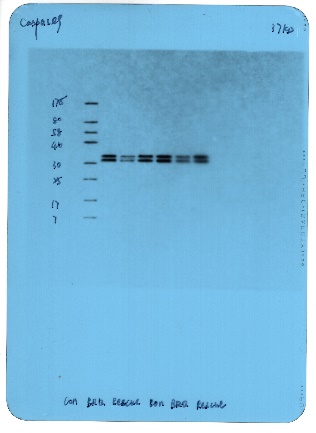

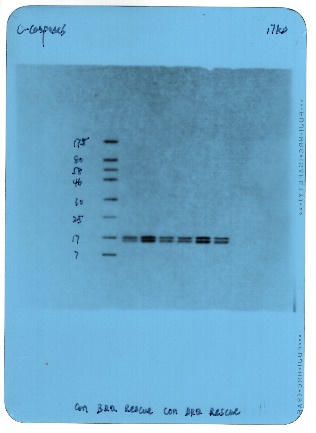

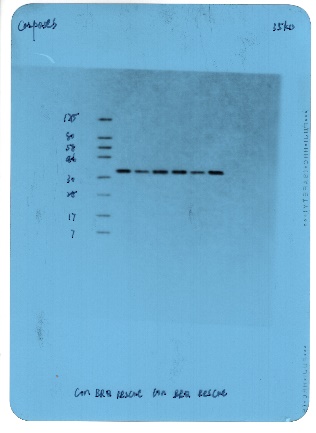

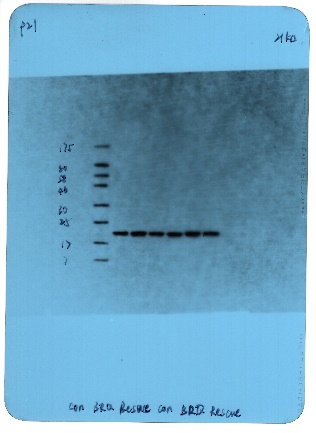

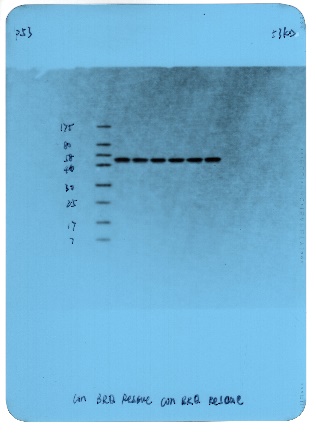

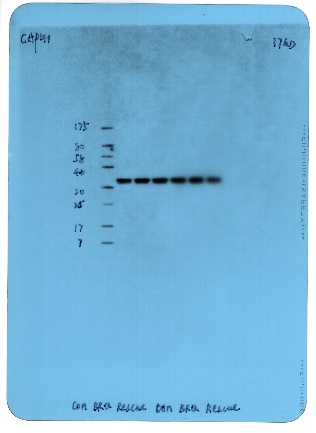


FIG2


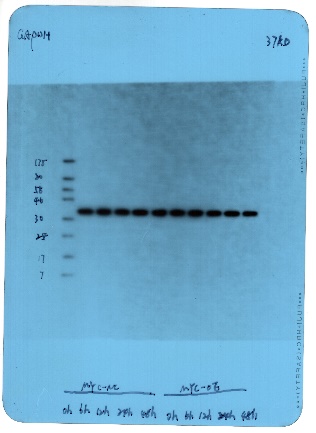

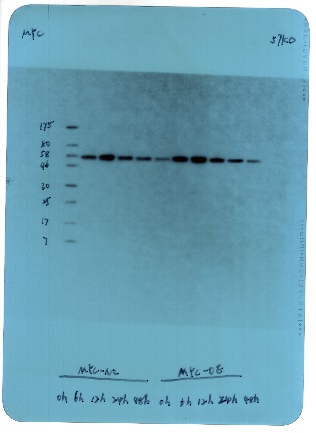

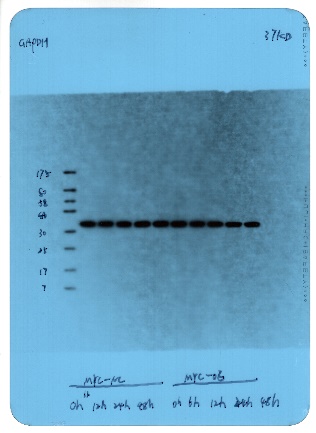

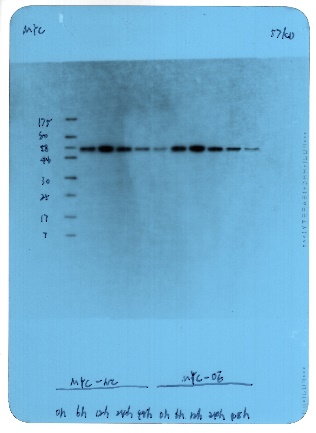

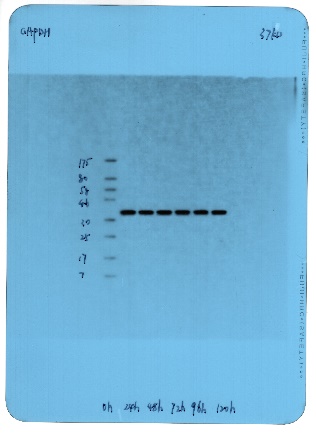

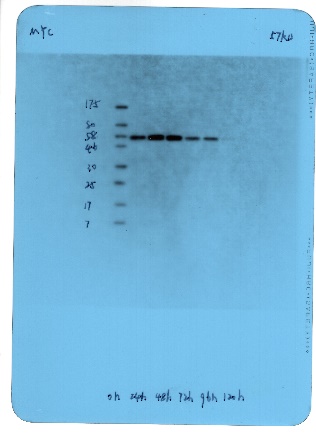

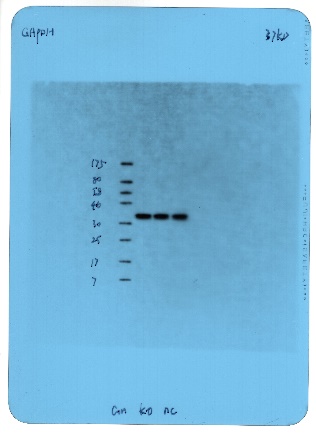

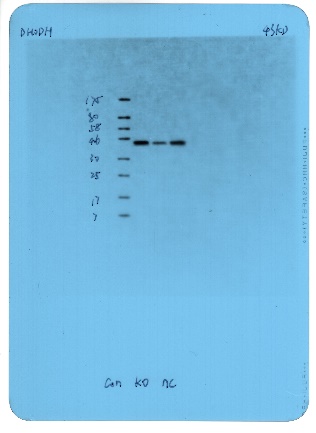

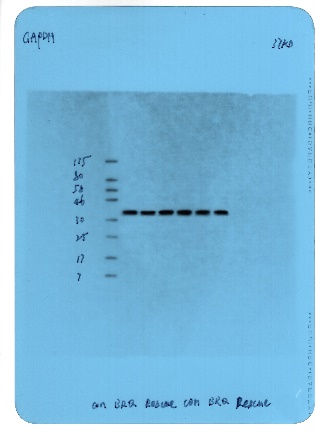

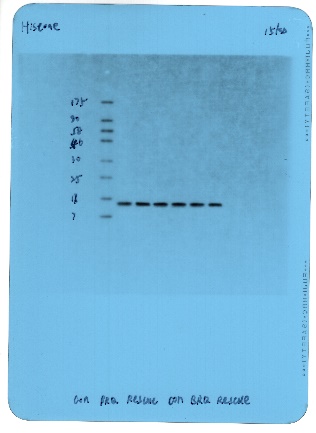

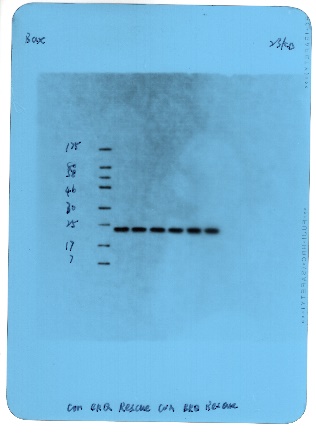

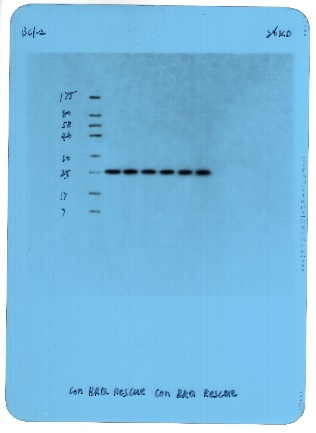

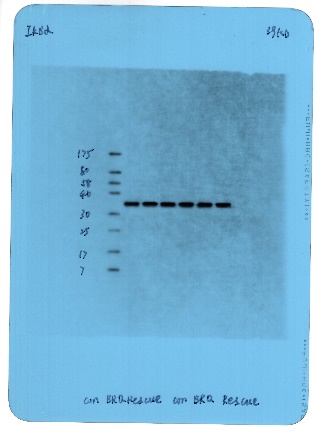

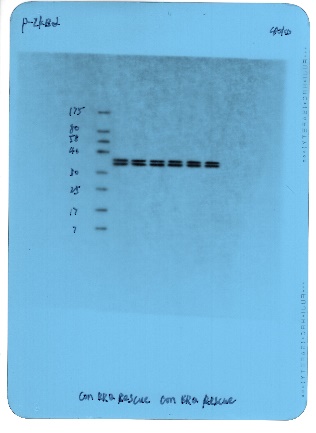

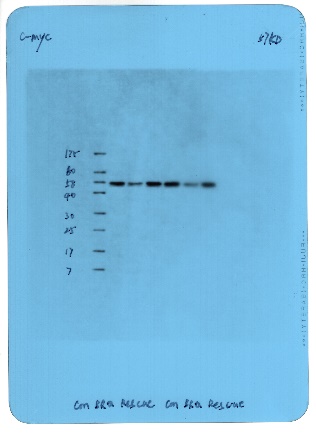

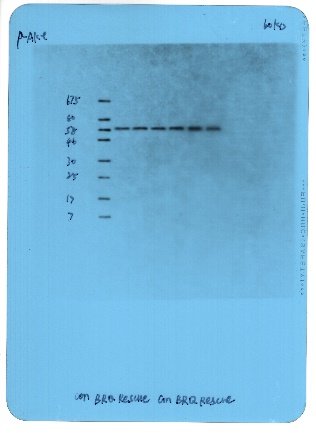

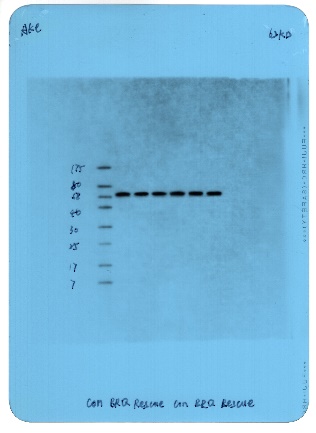

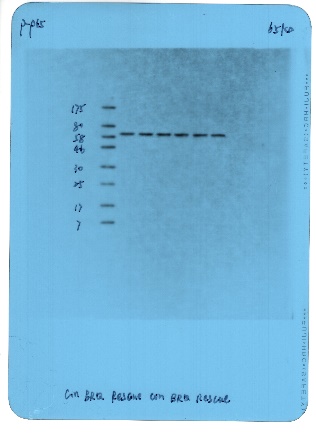

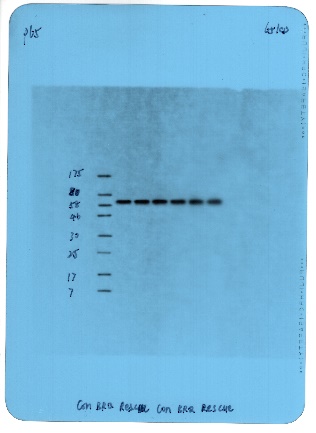

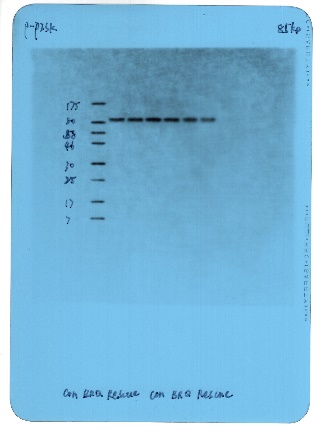

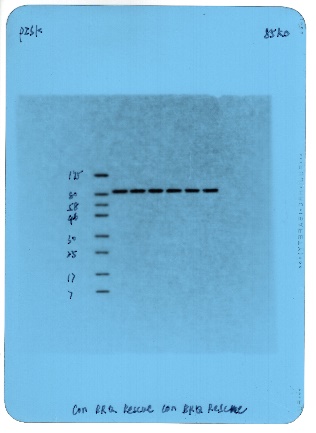

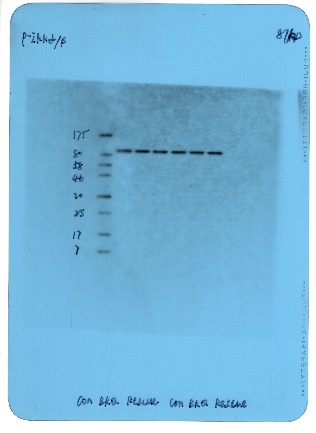

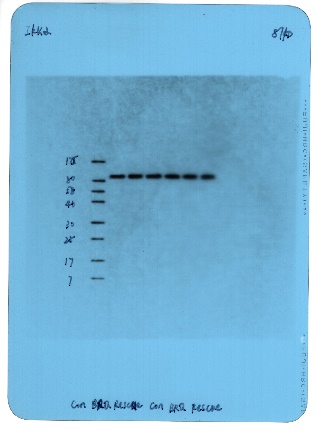

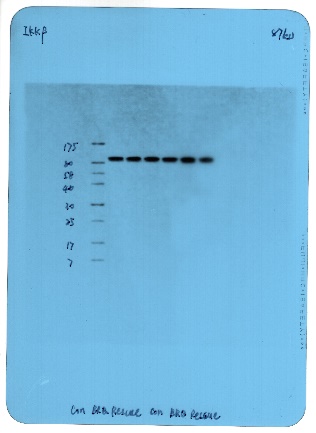


FIG4


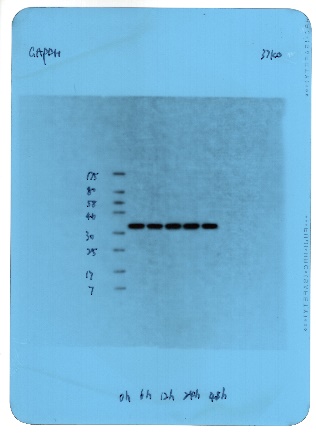

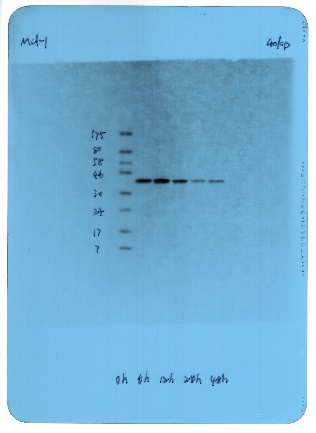

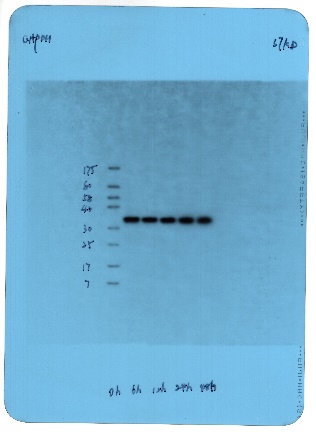

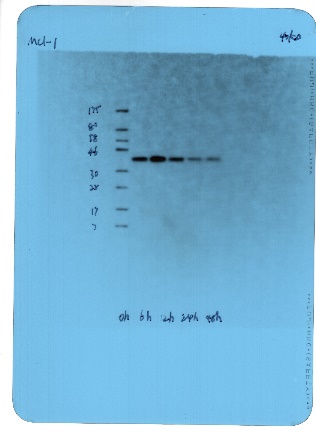

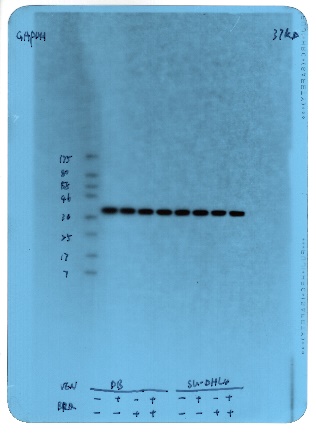

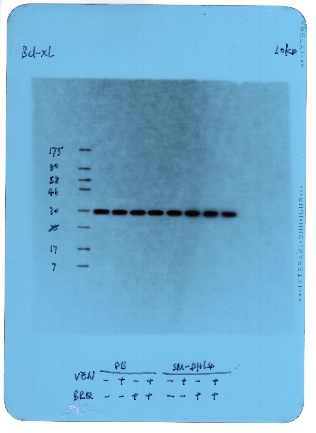

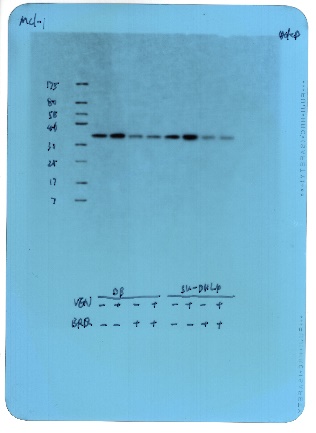

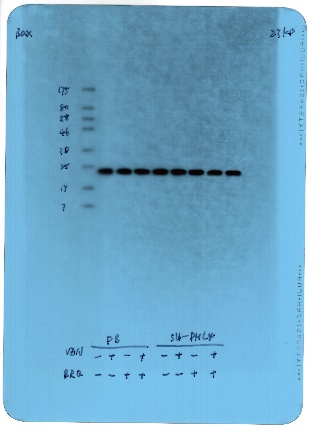

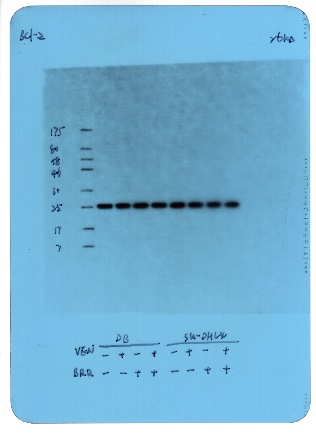

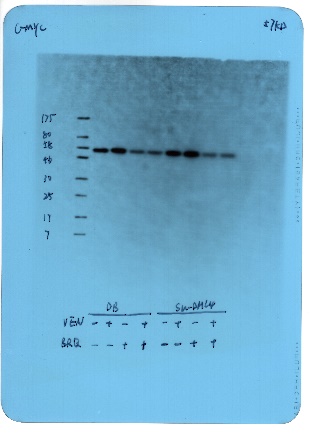

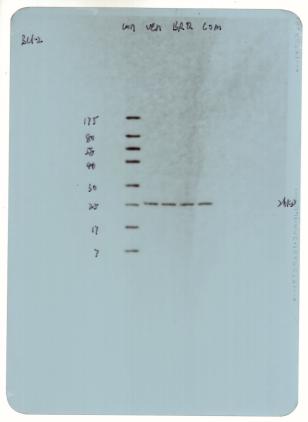

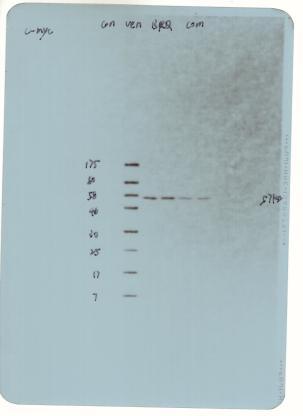


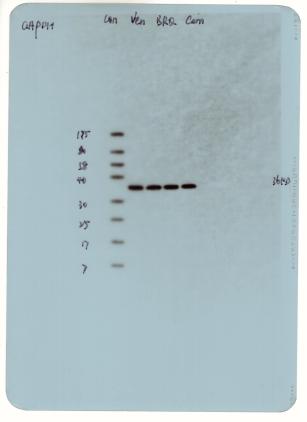

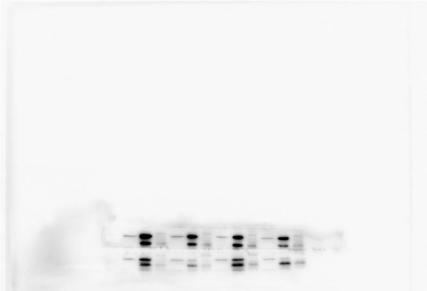

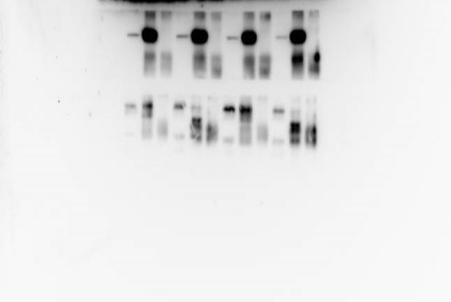


FIG6


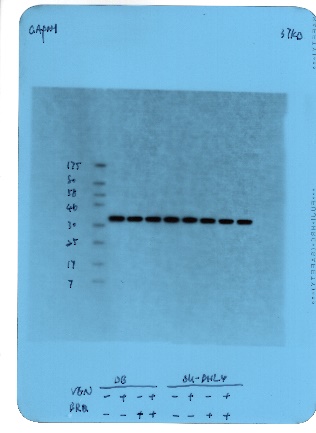

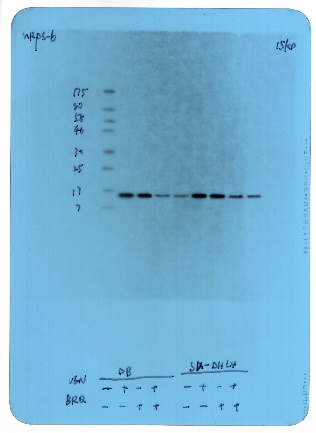

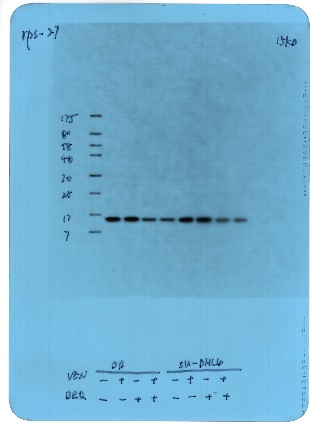

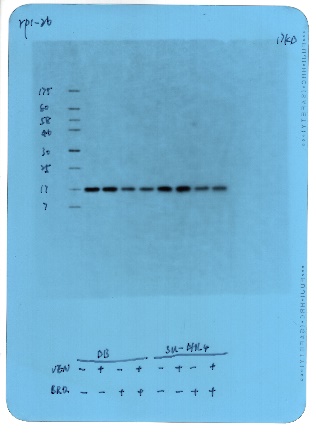

Supplement: Supplementary file 2 — Supplementary Material 2 [file 12885_2024_12534_MOESM2_ESM.docx]
