# Supplementary material for: Targeted inhibition of DHODH is synergistic with BCL2 blockade in HGBCL with concurrent MYC and BCL2 rearrangement
Source: BMC Cancer. 2024 Jun 25;24:761. doi: 10.1186/s12885-024-12534-w (PMC11197201; doi:10.1186/s12885-024-12534-w)
Supplement: Supplementary file 3 — Supplementary Material 3 [file 12885_2024_12534_MOESM3_ESM.docx]

**Supplementary Table 1. Primer sequences for real-time PCR.**

| primer | Primer-F | Primer-R |
| --- | --- | --- |
| MYC | 5’-CATCAGCACAACTACGCAGC | 3’-GCTGGTGCATTTTCGGTTGT |
| GAPDH | 5’-GTCTCCTCTGACTTCAACAGCG | 3’-ACCACCCTGTTGCTGTAGCCAA |
| RPL26 | 5’-GGCTAATGGCACAACTGTCCAC | 3’-GGCGAGATTTGGCTTTCCGTTC |
| RPS27 | 5’-AAGAAACGCCTGGTGCAGAGCC | 3’-TGTAGGCTGGCAGAGGACAGTG |
| MRPS6 | 5’-CGCTTCCTTATAGGATCTCTGCC | 3’-GAGACAAGTGCTCCACCATGCT |
| MCL-1 | 5’-CCAAGAAAGCTGCATCGAACCAT | 3’-CAGCACATTCCTGATGCCACCT |

**Supplementary Table 2. CI value of venetoclax and BRQ for 24,48,72 hours in DB cells and SU-DHL4 cells**

| Hours | CI- | Venetoclax-5 nM | Venetoclax-10 nM | Venetoclax-20 nM | Venetoclax-40 nM | Repeats |
| --- | --- | --- | --- | --- | --- | --- |
| DB-24 | BRQ-500 nM | 0.8904 ± 0.08839 | 1.009 ± 0.08902 | 0.7934 ± 0.08029 | 0.9087 ± 0.07761 | 3 |
| - | BRQ-1000 nM | 0.9621 ± 0.08701 | 0.9833 ± 0.1165 | 0.7702 ± 0.08846 | 0.9416 ± 0.1162 | 3 |
| - | BRQ-2000 nM | 0.9944 ± 0.1054 | 0.9717 ± 0.1014 | 0.7977 ± 0.102 | 0.8503 ± 0.1627 | 3 |
| - | BRQ-4000 nM | 0.9524 ± 0.06697 | 0.8158 ± 0.044 | 0.7843 ± 0.07383 | 0.7604 ± 0.1034 | 3 |
| DB-48 | BRQ-500 nM | 0.6434 ± 0.05395 | 0.6965 ± 0.06191 | 0.5614 ± 0.06624 | 0.6004 ± 0.05843 | 3 |
| - | BRQ-1000 nM | 0.698 ± 0.01372 | 0.7396 ± 0.07249 | 0.666 ± 0.09234 | 0.6584 ± 0.02548 | 3 |
| - | BRQ-2000 nM | 0.7618 ± 0.05879 | 0.7259 ± 0.08449 | 0.6719 ± 0.1132 | 0.6756 ± 0.1094 | 3 |
| - | BRQ-4000 nM | 0.6998 ± 0.07575 | 0.8017 ± 0.08767 | 0.7196 ± 0.07461 | 0.7345 ± 0.06861 | 3 |
| DB-72 | BRQ-500 nM | 1.144 ± 0.3158 | 1.155 ± 0.2301 | 1.084 ± 0.1732 | 1.153 ± 0.2521 | 3 |
| - | BRQ-1000 nM | 1.25 ± 0.2647 | 1.259 ± 0.2435 | 1.3 ± 0.2387 | 1.199 ± 0.2442 | 3 |
| - | BRQ-2000 nM | 1.292 ± 0.3037 | 1.333 ± 0.2549 | 1.319 ± 0.1971 | 1.227 ± 0.2072 | 3 |
| - | BRQ-4000 nM | 1.272 ± 0.3237 | 1.3 ± 0.3114 | 1.191 ± 0.2153 | 1.084 ± 0.1935 | 3 |
| SU-DHL4-24 | BRQ-500 nM | 0.9001 ± 0.08833 | 1.067 ± 0.05121 | 0.7085 ± 0.03846 | 0.8787 ± 0.09149 | 3 |
| - | BRQ-1000 nM | 0.8048 ± 0.1159 | 0.9769 ± 0.05567 | 0.658 ± 0.04344 | 0.8333 ± 0.1498 | 3 |
| - | BRQ-2000 nM | 0.9494 ± 0.1114 | 1.018 ± 0.07776 | 0.779 ± 0.02462 | 0.8546 ± 0.1076 | 3 |
| - | BRQ-4000 nM | 0.9137 ± 0.03033 | 0.9189 ± 0.1073 | 0.7869 ± 0.09034 | 0.8544 ± 0.1304 | 3 |
| SU-DHL4-48 | BRQ-500 nM | 0.9284 ± 0.09252 | 0.9108 ± 0.1292 | 0.7866 ± 0.09529 | 1.055 ± 0.2448 | 3 |
| - | BRQ-1000 nM | 0.9596 ± 0.1782 | 1 ± 0.1778 | 0.8449 ± 0.1339 | 1.09 ± 0.2788 | 3 |
| - | BRQ-2000 nM | 0.8493 ± 0.05901 | 0.8792 ± 0.1011 | 0.7501 ± 0.04941 | 0.9794 ± 0.1388 | 3 |
| - | BRQ-4000 nM | 0.8739 ± 0.1125 | 0.9329 ± 0.1521 | 0.8589 ± 0.1313 | 0.9519 ± 0.2102 | 3 |
| SU-DHL4-72 | BRQ-500 nM | 1.217 ± 0.1469 | 0.977 ± 0.1128 | 0.9686 ± 0.1051 | 0.9624 ± 0.108 | 3 |
|  | BRQ-1000 nM | 1.053 ± 0.0355 | 0.8953 ± 0.1028 | 0.8582 ± 0.1063 | 1.007 ± 0.1051 | 3 |
|  | BRQ-2000 nM | 1.175 ± 0.08582 | 0.9501 ± 0.146 | 0.9418 ± 0.1021 | 1.054 ± 0.143 | 3 |
|  | BRQ-4000 nM | 1.212 ± 0.01565 | 0.912 ± 0.1561 | 0.9525 ± 0.1112 | 0.9659 ± 0.1502 | 3 |
