# Supplementary material for: Targeted inhibition of DHODH is synergistic with BCL2 blockade in HGBCL with concurrent MYC and BCL2 rearrangement
Source: BMC Cancer. 2024 Jun 25;24:761. doi: 10.1186/s12885-024-12534-w (PMC11197201; doi:10.1186/s12885-024-12534-w)
Supplement: Supplementary file 4 — Supplementary Material 4 [file 12885_2024_12534_MOESM4_ESM.docx]

**
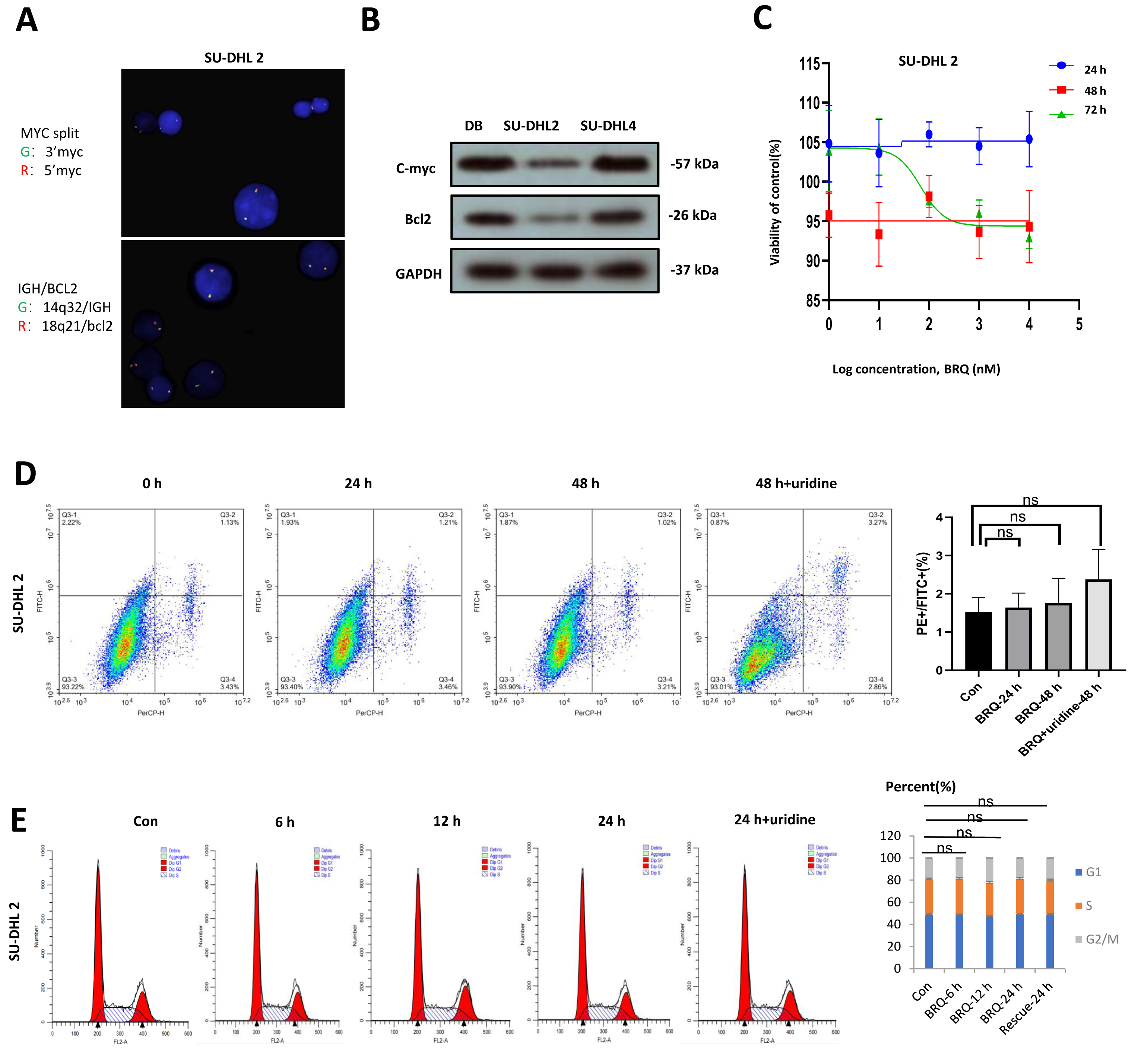
**

**Supplementary Figure 1. A**) FISH analysis of SU-DHL2 cells confirmed no c-MYC and BCL2 translocation. **B)** Protein levels of c-MYC and BCL-2 from DB, SU-DHL2 and SU-DHL4 cells. **C)** Dose-dependent effects of BRQ on the viability of SU-DHL-2 cell. The cells were treated with DMSO as the control or 1~10000 nM BRQ for 24 to 72 hours. The cell viability was calculated by the formula: Cell viability (%) = [OD (drug+) - OD (Blank)] / [OD (drug-) - OD (Blank)] × 100% at 24h, 48h and 72h. Data was normalized as 100% by the formula: OD (drug-) - OD (Blank). The x-axis log 0 means 1 nM BRQ. **D**) Flow cytometry cell apoptosis assays were used in SU-DHL2 cells with DMSO (con), 5 μM BRQ and 5 μM BRQ plus 1000 µM uridine (rescue) for 24 and 48 hours. Significance was achieved by two-way ANOVA followed by Bonferroni post-hoc analysis. *, P < 0.05, **, P < 0.01, ***, P < 0.001. **E)** Cell cycle analysis in BRQ-treated control cells by flow cytometry using propidium iodide (PI). SU-DHL-2 cells were treated with DMSO or 5 μM BRQ for 6, 12, and 24 hours and 5 μM BRQ plus 1000 µM uridine for 24 hours. The percentage of cells in G2/M phases is indicated in the histogram. ***, P < 0.001.

**
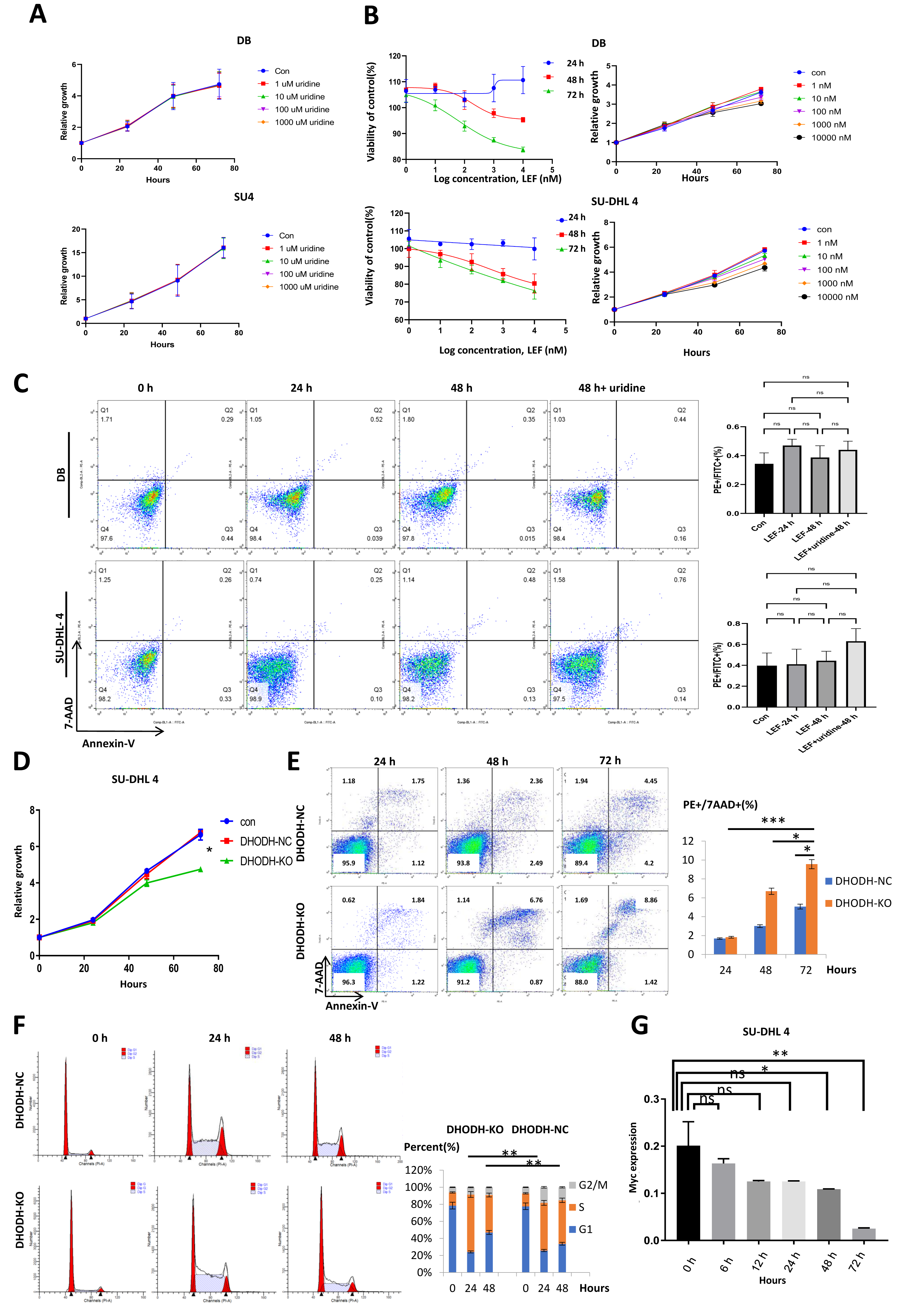
**

**Supplementary Figure 2. A)** Dose-dependent effects of uridine on the viabilities of DB and SU-DHL-4 cells. The cells were treated with DMSO as the control or 1~1000 μM uridine for 24 to 72 hours. **B)** Dose-dependent effects of leflunomide on the viabilities of DB and SU-DHL-4 cells. The cells were treated with DMSO as the control or 1~10000 nM LEF for 24 to 72 hours. **C)** Flow cytometry cell apoptosis assays were used in DB and SU-DHL4 cells with DMSO (con), 10 μM LEF and 10 μM LEF plus 1000 µM uridine (rescue) for 24 and 48 hours. Significance was achieved by one-way ANOVA followed by Turkey’s test. *, P < 0.05, **, P < 0.01, ***, P < 0.001. **D)** The cell viability of the control, DHODH-KO, and DHODH-NC cells cultured in medium without uridine for 24, 48, and 72 hours. **E)** Apoptosis assay of DHODH-KO and DHODH-NC cells cultured in medium without uridine for 24 and 48 hours. The values shown represent the mean ± SE of 3 independent experiments. *, P < 0.05, **, P < 0.01. **F)** Cell cycle analysis of DHODH-KO and DHODH-NC cells cultured in medium without uridine for 24 and 48 hours. The values shown represent the mean ± SE of 3 independent experiments. **, P < 0.01. Con, untreated SU-DHL4 cells; DHODH-KO, DHODH-knockout SU-DHL4 cells; DHODH-NC, empty vector-expressing SU-DHL4 cells. **G)** Gene expression levels of Myc in DHODH-KO SU-DHL4 cells cultured in medium without uridine for the indicated hours.


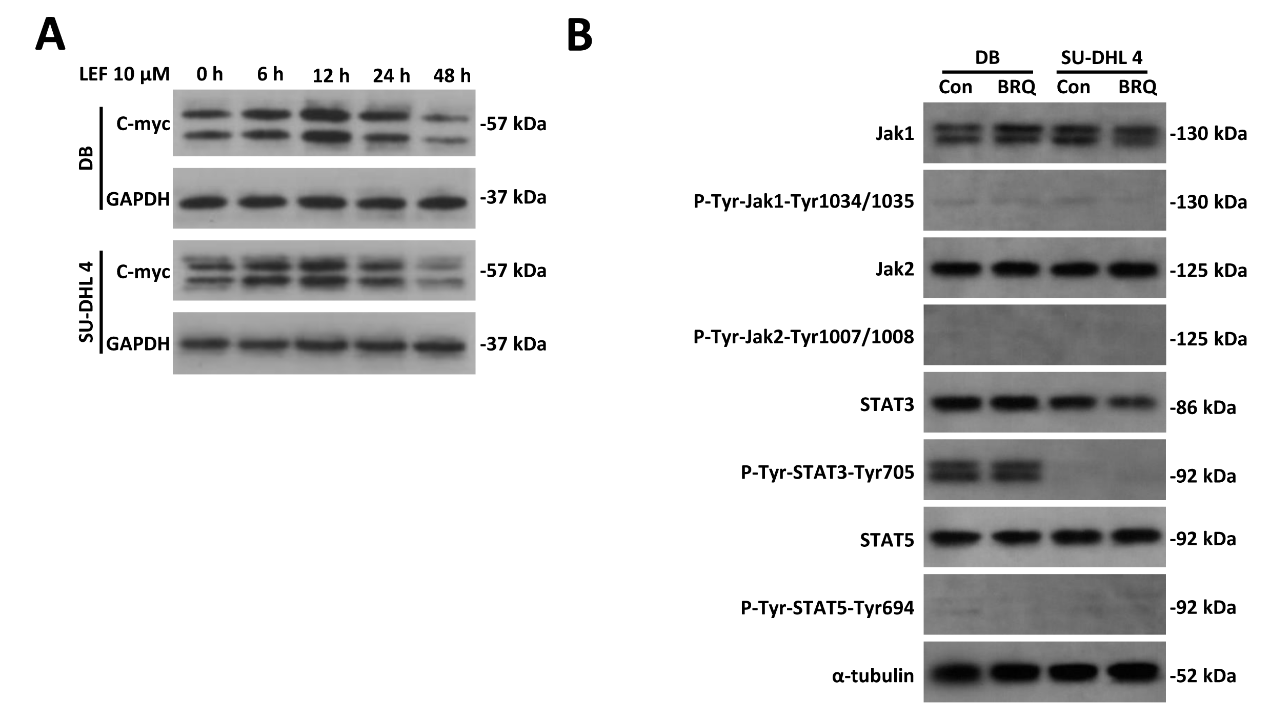


**Supplementary Figure 3. A)** The protein level of c-MYC in DB and SU-DHL4 cells treated with 10 μM LEF for 0h, 6h, 12h, 24h, and 48h. **B)** The protein levels of the JAK/STAT pathway in DB and SU-DHL4 cells treated with DMSO and 5 μM BRQ for 48 hours.

**
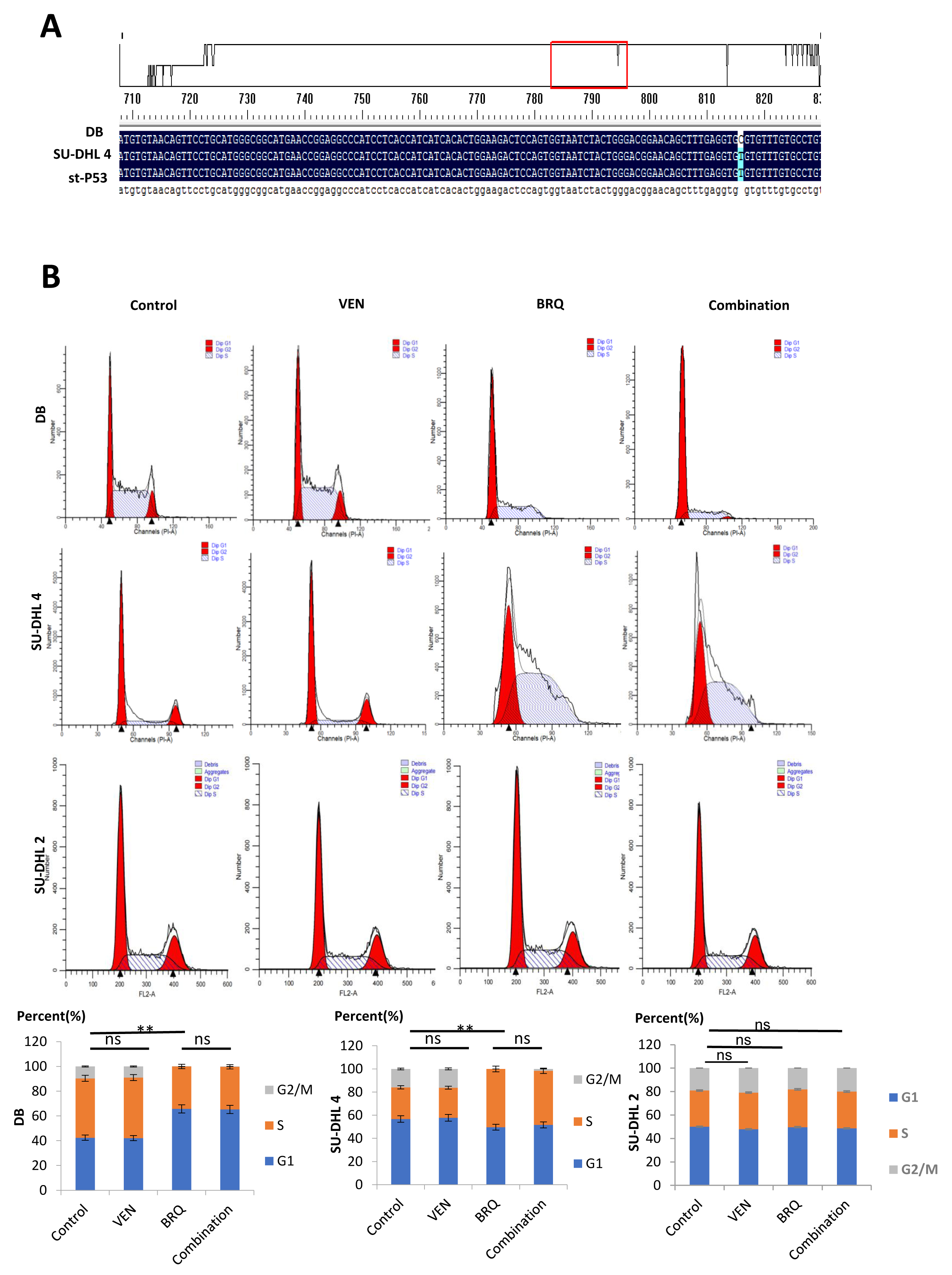
**

**Supplementary Figure 4.** **A**) Sanger sequence of P53 gene of DB and SU-DHL4 cells. **B**) Cell cycle analysis of DB, SU-DHL2 and SU-DHL4 cells after treatment with DMSO (Control), 20 nM venetoclax (VEN), 500 nM BRQ, or 20 nM venetoclax plus 500 nM BRQ (Combination) for 24 hours. The percentage of cells in G2/M phase is indicated in the histogram as the means ± SE of 3 independent experiments. *, P < 0.05, **, P < 0.01, ***, P < 0.001.


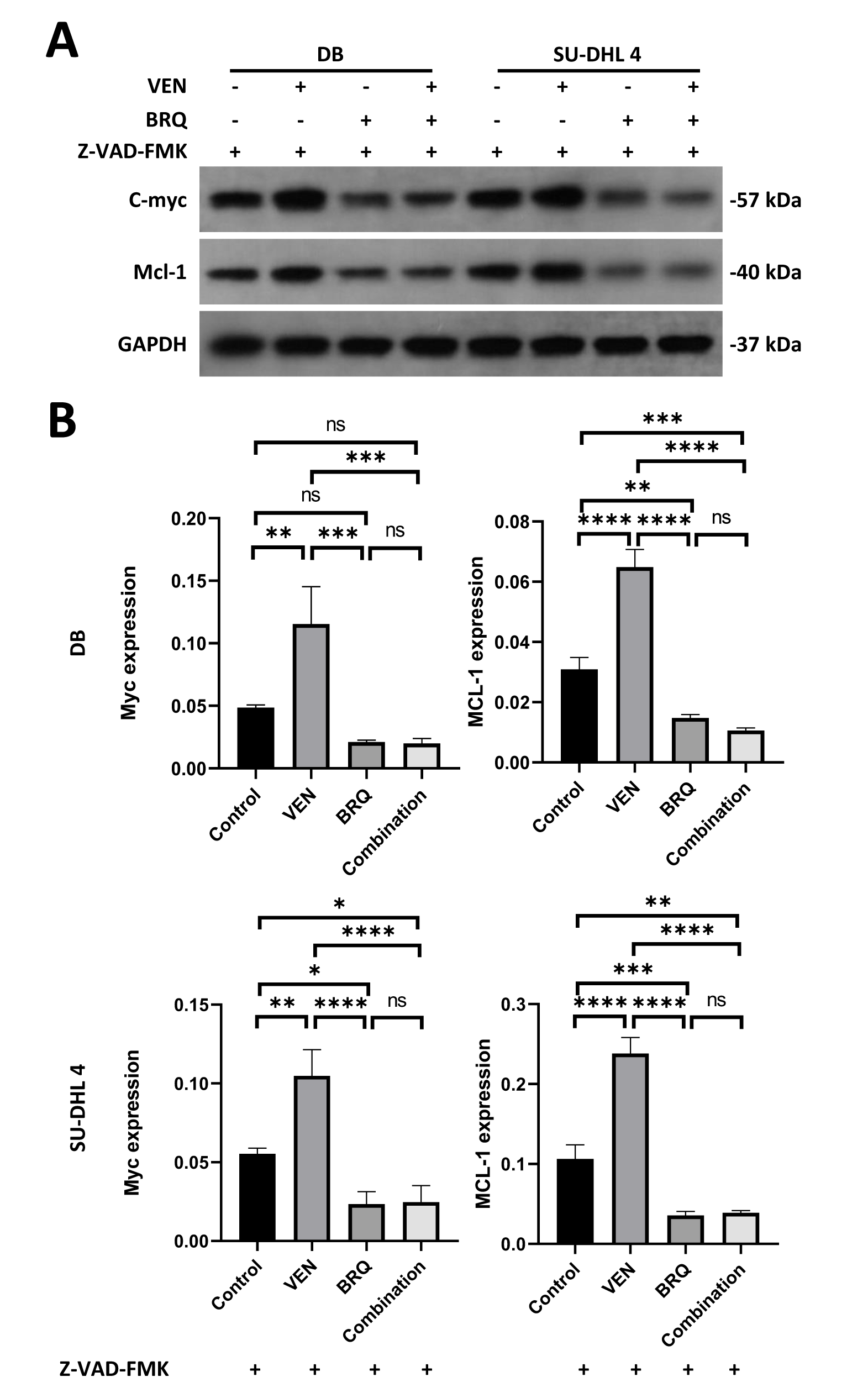


**Supplementary Figure 5. A)** The protein levels of c-MYC and MCL-1 in DB and SU-DHL4 cells treated with DMSO, 20 nM venetoclax, 500 nM BRQ, or 20 nM venetoclax plus 500 nM BRQ and the 200 μM caspase inhibitor z-VAF-FMK for 48 hours. **B)** Gene expression of MYC and MCL1 from DB and SU-DHL4 cells treated with DMSO, 20 nM venetoclax, 500 nM BRQ, or 20 nM venetoclax plus 500 nM BRQ and 200 μM caspase inhibitor z-VAF-FMK for 48 hours. The values were calculated from 3 independent experiments. Significance was achieved by one-way ANOVA followed by Turkey’s test. ****, P<0.0001, ***, P < 0.001, **, P < 0.01, *, P < 0.05.

**
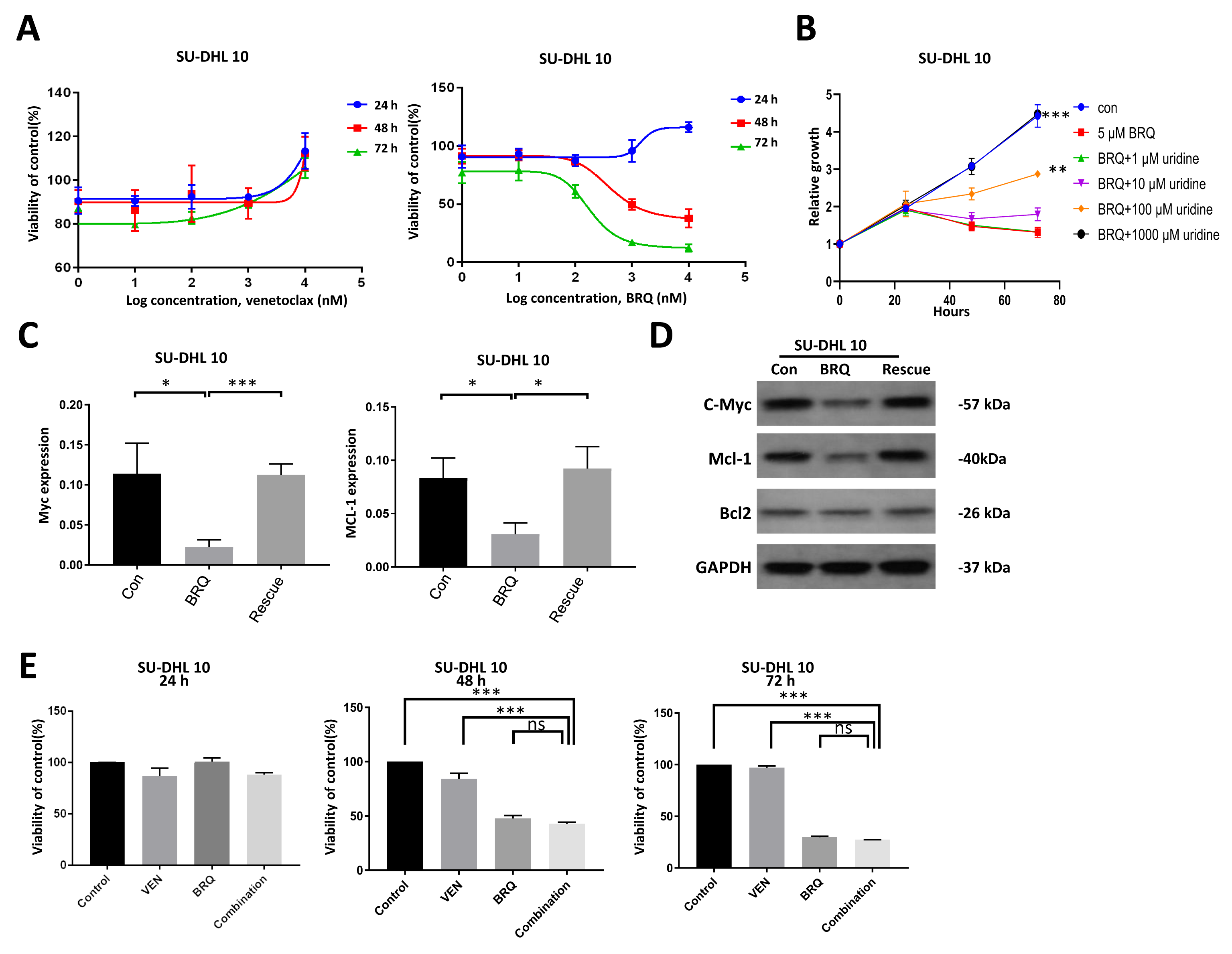
**

**Supplementary Figure 6. A)** Cell viability of SU-DHL10 cells treated with the indicated concentrations of venetoclax and BRQ for 24, 48, and 72 hours. **B)** Cell proliferation assay of SU-DHL10 cells treated with DMSO or 5 μM BRQ with or without uridine at the indicated time points (hours). The values are shown as the mean ± SE of 3 independent experiments. *, P < 0.05, **, P < 0.01, ***, P < 0.001. **C)** Gene expression of MYC and MCL1 downregulated by BRQ could be rescued by uridine. SU-DHL10 cells were incubated with DMSO, 5 μM BRQ, or 5 μM BRQ and 1000 µM uridine for 48 hours. The values are shown as the mean ± SE of 3 independent experiments. *, P < 0.05, ***, P < 0.001. **D)** The protein levels of c-MYC, MCL-1, and BCL-2 in SU-DHL10 cells were detected by western blotting after treatment with DMSO, 5 μM BRQ, and BRQ with 1000 µM uridine for 48 hours. **E)** The venetoclax and BRQ combination showed no synergistic repression effect on the viability of SU-DHL10 cells. Cell viability of SU-DHL10 cells treated with DMSO, 1 μM venetoclax, 500 nM BRQ, and combined venetoclax and BRQ for 48 hours. The values are shown as the mean ± SE of 3 independent experiments. *, P < 0.05, **, P < 0.01, ***, P < 0.001.


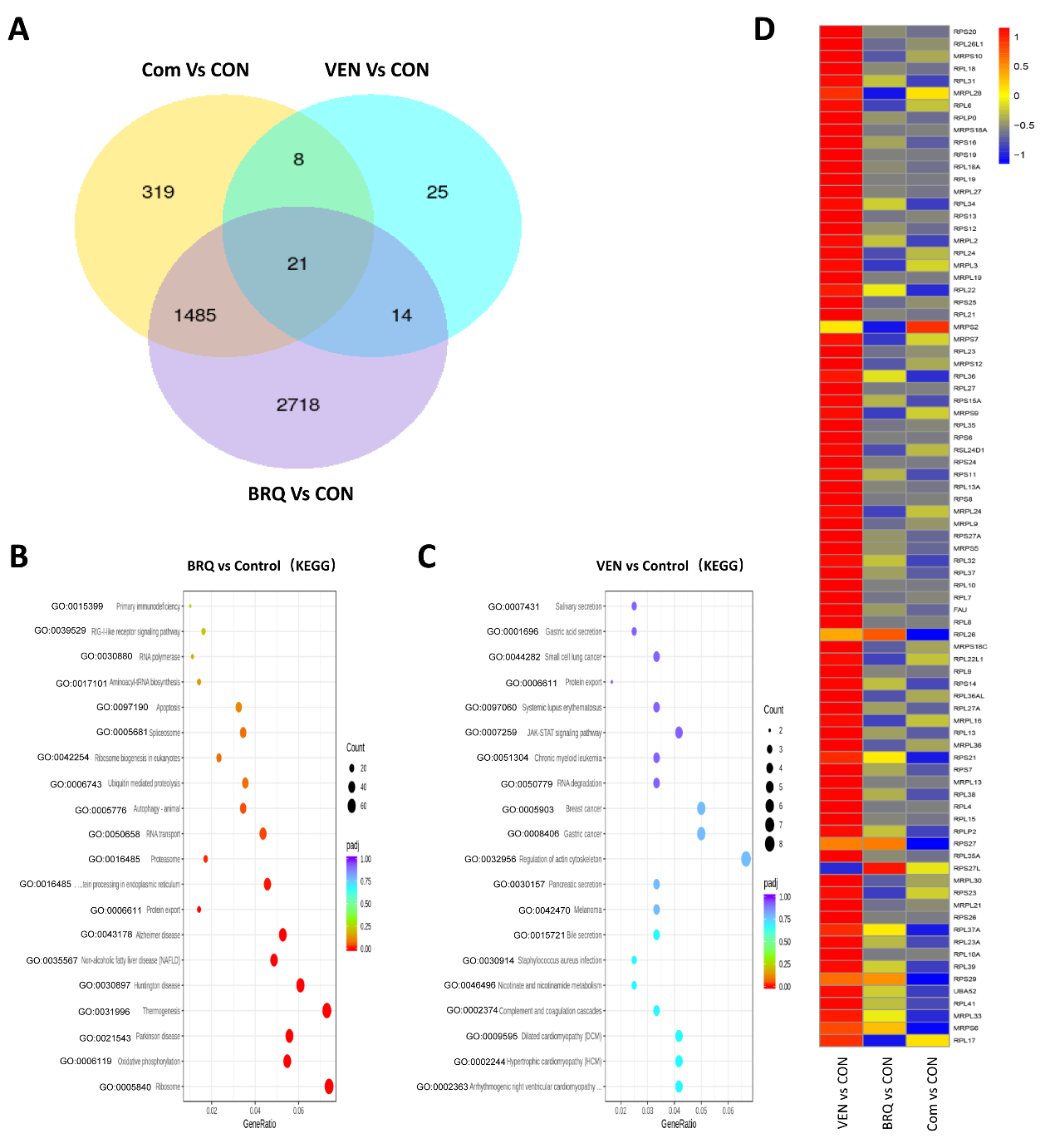


**Supplementary Figure 7.** **A)** Venn diagram showing the overlap of differentially expressed genes between different groups. **B)** GO enrichment analysis of SU-DHL4 cells treated with BRQ compared with DMSO (control). **C)** GO enrichment analysis of SU-DHL4 cells treated with venetoclax (VEN) compared with DMSO (Control). **D)** Heatmap of differentially expressed transcripts of the ribosome pathway. SU-DHL4 cells were treated with DMSO as a control, 500 nM BRQ, or 20 nM venetoclax as a monotreatment, and 500 nM BRQ plus 20 nM venetoclax as a combination treatment group for 48 hours.


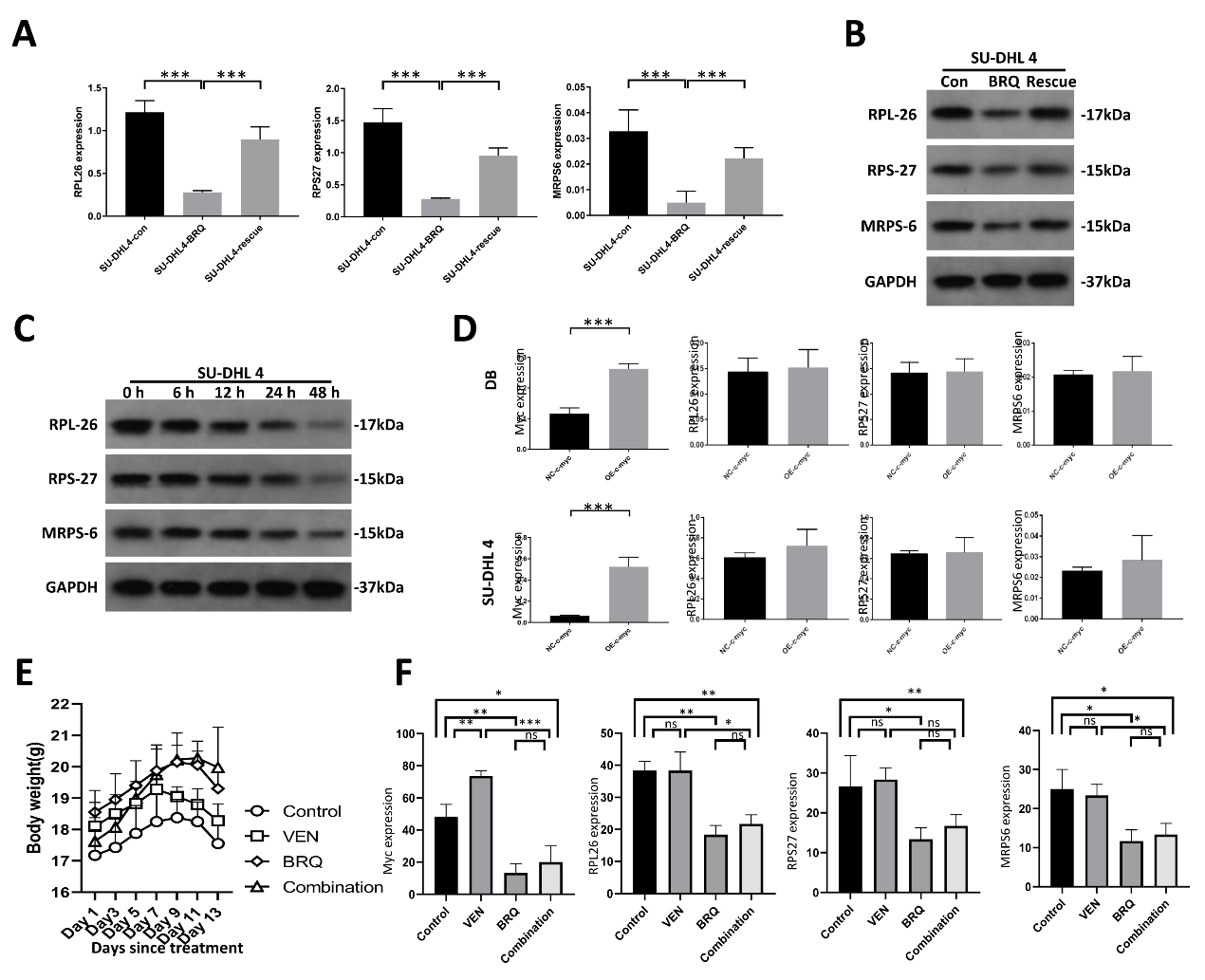


**Supplementary Figure 8. A)** Gene expression of RPL26, RPL27, and MRPS6 downregulated by BRQ could be rescued by uridine. SU-DHL4 cells were incubated with DMSO, 5 μM BRQ, or BRQ and 1000 µM uridine for 48 hours. The values are shown as the mean ± SE of 3 independent experiments. ***, P < 0.001. **B)** The protein levels of RPL26, RPL27, and MRPS6 in SU-DHL4 cells were detected by western blotting after treatment with DMSO, 5 μM BRQ, or BRQ and 1000 µM uridine for 48 hours. Data are shown as means ± standard error (SE). All the experiments were performed in triplicate. **C)** The protein levels of RPL-26, RPL-27, and MRPS-6 in SU-DHL4 cells were determined by western blot after 0, 6, 12, 24, and 48 hours of 5 μM BRQ treatment. **D)** Gene expression of MYC, RPL26, RPL27, and MRPS6 in MYC-NC and MYC-OE DB and SU-DHL4 cells. The values are shown as the mean ± SE of 3 independent experiments. ***, P < 0.001. **E)** Body weight curves in tumor-bearing NOD-SCID mice after SU-DHL4 cells inoculation. **F)** Quantification of c-MYC, RPL-26, RPL-27, and MRPS-6 positive cells in indicated treatment groups.
